# Divergent contribution of the MVA and MEP pathways to the formation of polyprenols and dolichols in Arabidopsis

**DOI:** 10.1042/BCJ20220578

**Published:** 2023-04-21

**Authors:** Agata Lipko, Cezary Pączkowski, Laura Perez-Fons, Paul D. Fraser, Magdalena Kania, Marta Hoffman-Sommer, Witold Danikiewicz, Michel Rohmer, Jaroslaw Poznanski, Ewa Swiezewska

**Affiliations:** 1 Institute of Biochemistry and Biophysics, Polish Academy of Sciences, Warsaw, Poland; 2 Department of Plant Biochemistry, Faculty of Biology, University of Warsaw, Warsaw, Poland; 3 School of Biological Sciences, Royal Holloway, University of London, Egham Hill, U.K.; 4 Institute of Organic Chemistry, Polish Academy of Sciences, Warsaw, Poland; 5 Université de Strasbourg/CNRS, Institut Le Bel, Strasbourg, France

**Keywords:** *Arabidopsis thaliana*, cross-talk, dolichol, MEP pathway, MVA pathway, polyprenol

## Abstract

Isoprenoids, including dolichols (Dols) and polyprenols (Prens), are ubiquitous components of eukaryotic cells. In plant cells, there are two pathways that produce precursors utilized for isoprenoid biosynthesis: the mevalonate (MVA) pathway and the methylerythritol phosphate (MEP) pathway. In this work, the contribution of these two pathways to the biosynthesis of Prens and Dols was addressed using an *in planta* experimental model. Treatment of plants with pathway-specific inhibitors and analysis of the effects of various light conditions indicated distinct biosynthetic origin of Prens and Dols. Feeding with deuteriated, pathway-specific precursors revealed that Dols, present in leaves and roots, were derived from both MEP and MVA pathways and their relative contributions were modulated in response to precursor availability. In contrast, Prens, present in leaves, were almost exclusively synthesized via the MEP pathway. Furthermore, results obtained using a newly introduced here ‘competitive’ labeling method, designed so as to neutralize the imbalance of metabolic flow resulting from feeding with a single pathway-specific precursor, suggest that under these experimental conditions one fraction of Prens and Dols is synthesized solely from endogenous precursors (deoxyxylulose or mevalonate), while the other fraction is synthesized concomitantly from endogenous and exogenous precursors. Additionally, this report describes a novel methodology for quantitative separation of ^2^H and ^13^C distributions observed for isotopologues of metabolically labeled isoprenoids. Collectively, these *in planta* results show that Dol biosynthesis, which uses both pathways, is significantly modulated depending on pathway productivity, while Prens are consistently derived from the MEP pathway.

## Introduction

Isoprenoids constitute a very large and diverse group of natural compounds and their structural and functional diversity is especially astonishing in plants. They include representatives of both primary (phytosterols, photosynthetic pigments, plastoquinone, plant hormones) and secondary (specialized) metabolites (e.g. volatile monoterpenes, defensive sesquiterpenes) which play vital roles in basic physiological processes in plants as well as in the interactions of plants with the environment [1,2]. The use of plant isoprenoids as pharmaceuticals, fragrances, flavors, colorants, and dietary supplements makes them the most commercially exploited group of plant-derived natural products [3–5]. Unraveling the details of the isoprenoid biosynthetic network is therefore of interest for basic studies and it is also critical for the construction of efficient platforms for their production [6–8].

**Figure 1.**
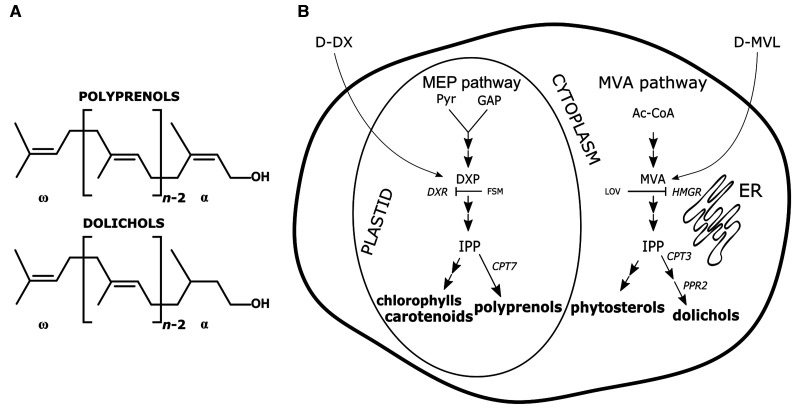
Polyisoprenoids in plant cell: (A) structures and (B) site of accumulation of polyprenols and dolichols. (**A**) Both terminal groups of polyisoprenoid alcohol are marked: C-terminal (ω) and hydroxylated (α), *n* corresponds to the number of isoprene units in the molecule. (**B**) organellar-specific accumulation of isoprenoid lipids is shown together with the simplified scheme of isoprenoid-generating metabolic routes — the mevalonate (MVA) pathway and the methylerythritol phosphate (MEP) pathway. Key enzymes producing polyisoprenoids in Arabidopsis are marked: *cis*-prenyltransferase 3 and −7 (CPT3 and −7) and polyprenol reductase 2 (PPRD2). Applied in this study deuteriated pathway-specific precursors (deoxyxylulose, D-DX, and mevalonate, D-MVL) as well as pathway-specific inhibitors (lovastatin, LOV, and fosmidomycin, FSM) are depicted.

Dols together with Prens are polyisoprenoid alcohols (Figure 1A). Their formation starts with an allylic initiator, either farnesyl diphosphate (FPP) or geranylgeranyl diphosphate (GGPP), which undergoes subsequent head-to-tail condensations with a specific number of isopentenyl diphosphate (IPP) molecules. This reaction is catalyzed by dedicated *cis*-prenyltransferases (CPTs) [9] and produces polyprenyl diphosphate, which then undergoes dephosphorylation to Pren and eventually its OH-terminal isoprene unit may be reduced to synthesize Dol [10,11].

Polyisoprenoids are ubiquitous in living organisms — bacteria synthesize mostly a single Pren (predominantly undecaprenol) while yeast, fungi and animal cells produce mixtures (‘families’) of Dols. Quite uniquely, plants accumulate families of both groups: Prens are found in green tissues, flowers and wood while Dols are found mainly in roots, although all tissues contain small amounts of both types of polyisoprenoids. A recent study has revealed that leaves of *Arabidopsis thaliana* contain a family of Prens composed of 9 to 11 isoprene units (Pren-9 to Pren-11) synthesized by CPT7. The Dol spectrum in *A. thaliana* (studied in hairy roots) comprises two main families: medium-length Dols (Dol-15, -16 and -17, synthesized by CPT3 [12]) and longer ones (Dol-20 to Dol-24, produced by CPT1 [13]), all of which are accompanied by traces of corresponding Prens [14]. The composition of polyisoprenoid mixtures is believed to be regulated by tissue-specific expression of dedicated CPT(s); in the Arabidopsis genome nine potential members of this multigene family have been identified [15].

The hydroxyl group in polyisoprenoid alcohols remains either free or phosphorylated or esterified with carboxylic acids. The role of polyisoprenoid phosphates is well documented. Dolichyl phosphates (Dol-P) are indispensable in protein *N*-glycosylation, *O*- and *C*-mannosylation and glycosylphosphatidyl inositol-anchor (GPI) synthesis in eukaryotic cells [16] while prenyl phosphates (Pren-P) play a similar role in protein modification and glycan biosynthetic pathways in bacteria [17]. Malfunctions in the Dol metabolism lead to severe dysfunctions in mammals, manifesting in humans as inherited diseases called Congenital Disorders of Glycosylation [18]. In plants, a shortage of Dols is lethal due to defects in both gametophyte and sporophyte development [11]. In contrast, Prens do not seem indispensable for plant viability, though they might affect plant fitness via exerting dynamic control over thylakoid membranes [19]. Additionally, basing on results obtained for model membranes [20–22] polyisoprenoids are postulated to affect the physicochemical properties (e.g. permeability, fluidity) of biological membranes. They are also proposed to serve as cellular reactive oxygen species (ROS) scavengers [23–25].

Isopentenyl diphosphate (IPP) and its isomer — dimethylallyl diphosphate (DMAPP) — are building blocks from which isoprene units are formed. IPP and DMAPP are initially condensed to produce all-*trans*-(*E*)-prenyl diphosphates: farnesyl (FPP, C_15_) and geranylgeranyl (GGPP, C_20_) diphosphates, which are then used as initiators to produce various isoprenoids. Two pathways engaged in IPP/DMAPP biosynthesis are known. The mevalonate (MVA) pathway is found in animals, fungi and archaebacteria and the 2-*C*-methyl-D-erythritol 4-phosphate (MEP) pathway is characteristic for protozoa, most of eubacteria and algae. In this context, higher plants, utilizing both pathways in parallel, are unique. In plant cells the MVA pathway is localized in the cytoplasm/ER and possibly also in peroxisomes [26] while the MEP pathway enzymes are sequestered in plastids [27,28] (Figure 1B). Despite the compartmentalization, an exchange of intermediates between the pathways (‘cross-talk’) takes place, although its regulation and mechanism still remain largely unknown [2]. The majority of isoprenoids are considered to derive either from the MVA pathway (sesquiterpenoids, triterpenoids, e.g. phytosterols) or from the MEP pathway (monoterpenoids, diterpenoids, tetraterpenoids, e.g. carotenoids), but in many cases, a contribution from the alternative pathway occurs conditionally, depending on the plant species, tissue, developmental stage or physiological conditions [29,30]. The exchange of (yet unidentified) intermediate(s) between plastids and the cytoplasm is sometimes so substantial that it leads to the formation of isoprenoid compounds of mixed MVA/MEP origin and, based on data obtained in hairy roots, Dols are examples of such ‘mosaic’ isoprenoids [31]. Interestingly, the relative contribution of the MEP pathway to medium-length dolichols (Dol-14 to Dol-17) was clearly increased by osmotic stress [32]. This observation is in line with literature data (summarized in ref. [2]) suggesting that cross-talk in the MVA/MEP pathway is prone to modulation by environmental clues.

Very few studies on the MVA/MEP cross-talk have been performed on whole plants [30,33,34], the vast majority employed instead technically convenient cell or tissue cultures or detached organs. Consequently, exploration of the cooperation of isoprenoid-generating routes in intact plants is still required.

In this report, the contribution of both pathways to Dols and Prens biosynthesis in roots and leaves of Arabidopsis plants is dissected. The effects of pathway-specific inhibitors, light and exogenous pathway-specific metabolic precursors were investigated. Comparison of the deuteriation patterns together with the effects of inhibitor and light treatment led to the conclusion that Prens in plants are predominantly derived from the MEP pathway. In contrast, the biosynthetic origin of Dols appeared to involve both the MEP and the MVA pathway — in this respect our *in planta* study fully confirmed previous data obtained from hairy root cultures. Furthermore, the results of labeling suggest that access of the MEP pathway enzymes to exogenous deoxyxylulose (DX) — the MEP precursor used in this study — is somehow restricted and exogenous DX is to a large extent metabolized by a separate intracellular sub-route of the MEP pathway. Our data shed new light on the functioning of the isoprenoid network in plant leaves and roots and they provide a novel methodology for quantitative metabolic labeling.

## Materials and methods

### Chemicals

(5,5-^2^H_2_)-1-Deoxy-D-xylulose (D-DX) was synthesized as described earlier [35]. (±)-((6,6,6-^2^H_3_)Methyl)mevalonolactone (D-MVL) was purchased from EQ Laboratories GmbH (Augsburg, Germany) while their counterparts of natural ^2^H and ^13^C abundance, further on called natural isotope abundance precursors, were from Omicron Biochemicals (U.S.A.) for DX and from Avantor Performance (Gliwice, Poland) for (±)MVL. Lovastatin was purchased from Merck (Warsaw, Poland) and fosmidomycin, sodium salt — from ThermoFisher Scientific (Warsaw, Poland). Pren standards (Pren-19 and Pren mixture) were obtained from the Collection of Polyprenols IBB PAS (Warsaw, Poland). Components of hydroponic culture medium were purchased from Chempur (Piekary Slaskie, Poland).

### Plant material

*Arabidopsis thaliana* accession Columbia-0 plants (Nottingham Arabidopsis Stock Center, U.K.) were used in all the experiments.

### Plant growth conditions

Plants were grown in a growth chamber for 5 weeks at a short-day (SD, 8 h/16 h day/night) photoperiod (130–150 µmol m^−2^ s^−1^, 28/23°C day/night and 65% relative humidity). When indicated, plants were grown under long-day (LD, 12 h/12 h day/night) or continuous light (CL) conditions.

#### Semi-sterile hydroponic culture

Semi-sterile hydroponic culture was conducted using a hydroponic system (Araponics, Liege, Belgium). These plants were used for the analysis of the effects of inhibitors and different photoperiods on the accumulation of selected isoprenoids. Seeds were surface sterilized with an aqueous solution of 5% calcium hypochlorite for 8 min, placed on agar (0.7%) in the seed holders, covered with a transparent lid and stratified for 2 days (4°C) in the darkness. The lid was removed after 7 days. Once a week the medium (1.7 L) was replaced with a new batch.

#### Sterile hydroponic culture

Sterile hydroponic culture was used for metabolic labeling experiments. The home-designed growth system comprised a Magenta plant culture box (Sigma–Aldrich, Poland) with a plastic lid (holes in the lids were plugged with cotton) and stainless steel supporters. Surface sterilized seeds were sown on stainless steel wire mesh (1.2 mm aperture, 0.3 mm wire diameter, El-Decor, Cracow, Poland) cut to fit the wire supporters and covered with a thin (∼1 mm) layer of 0.7% agar. Seeds were then stratified for 2 days (4°C) in the darkness and transferred to the growth chamber. After 5 days the mesh with seedlings was transferred to the plant culture box containing approximately the hydroponic medium (180 ml) and placed on the wire supporter.

### Composition of hydroponic medium

The hydroponic growth medium (Gibeaut's solution) contained the following macroelements: 1.50 mM Ca(NO_3_)_2_, 1.25 mM KNO_3_, 0.75 mM MgSO_4_, 0.50 mM KH_2_PO_4_, 0.1 mM Na_2_O_3_Si, 72 mM Fe–EDTA, 50 µM KCl, and microelements: 50 µM H_3_BO_3_, 10 µM MnSO_4_, 2 µM ZnSO_4_, 1.5 µM CuSO_4_, 0.075 mM (NH_4_)_6_Mo_7_O_24_ [36].

### Inhibitor studies

Five-week-old plants grown under SD conditions in semi-sterile hydroponic culture were transferred to hydroponic medium supplemented with an ethanolic solution (0.5 ml) of lovastatin (LOV) or aqueous solution of fosmidomycin sodium salt (FSM) to the final concentrations of 100 and 400 µM, respectively. After 72 h leaves and roots were separately collected, frozen in liquid nitrogen and stored at −80°C for further analysis. Data for inhibitor-treated plants were compared with control (untreated) plants, in the case of LOV-treated plants the control growth medium was supplemented with ethanol (0.5 ml).

### Metabolic labeling

Sterile growth medium was supplemented with the indicated metabolic precursor(s): deuteriated or natural isotopic abundance MVL (that is in equilibrium with mevalonate and even more easily incorporated *in vivo* than mevalonate [37,38]) or DX at the final concentrations of 1 mM or 0.5 mM, respectively. Plants were grown at SD conditions and after 5 weeks roots and leaves were separately collected, lyophilized and stored at −80°C for further analysis. Prens were additionally analyzed in the leaves of plants grown in the presence of 0.5 mM D-DX for 4 weeks, and for further 24 or 48 h after replacement of the medium by fresh medium containing also 0.5 mM D-DX. Leaves were harvested and stored as above.

### Extraction and analysis of selected isoprenoids

#### Quantitative analysis

For quantitative analysis of total pools of polyisoprenoids [39] and phytosterols [13] crude lipid extracts isolated from leaves (∼2 g) and roots (∼0.8 g), respectively, were subjected to alkaline hydrolysis. Pren-19 (10 µg) and cholestanol (50 µg) were used as internal standards. Quantification of polyisoprenoid alcohols was performed with the aid of HPLC/UV [31] using a ZORBAX XDB-C18 (4.6 × 75 mm, 3.5 µm) reverse phase column (Agilent, U.S.A.). Content of phytosterols, analyzed as free alcohols, was estimated using a GC apparatus (7890A, Agilent Technologies, U.S.A.) equipped with a FID detector [14]. It should be kept in mind that protocol of isolation of polyisoprenoids and phytosterols employed here does not permit to elucidate the content of either their esters or saccharide-linked derivatives. Plastidial pigments were isolated for quantitative analysis [40] from fresh tissue (∼100 mg). Leaves were randomly chosen from the plant rosette or, when indicated, only three youngest leaves from the rosette center were analyzed (young leaves) (https://protocols.scienceexchange.com/protocols/leaf-numbering-for-experiments-on-long-distance-signalling-in-arabidopsis). Content of chlorophylls and carotenoids were determined in acetone leaf extract by the spectrophotometric method, absorption was measured at the wavelengths of 662 nm, 645 nm and 470 nm for chlorophyll a, chlorophyll b and total carotenoids, respectively; pigment content was calculated as described previously [41]. Results of the quantitative analysis of all isoprenoids are presented as means of at least three independent biological replicates ± SD.

#### Analysis of deuterium incorporation

For structural elucidation of the metabolically labeled polyisoprenoid alcohols, phytosterols and sterol precursors, leaf (∼0.8 g) root (∼300 mg) tissue was used. Isolation was performed as described earlier [39] with modifications. Briefly, leaf isoprenoids were purified from the total unsaponifiable lipids by preparative thin layer chromatography (silica gel plate, thickness 0.5 mm, Merck). The plate was developed in dichloromethane (two migrations), air-dried, sprayed with berberine sulfate (1% in ethanol) and visualized under UV light (360 nm). Bands corresponding to the mobility of standards were scraped, products were eluted from the silica gel with chloroform. The fraction containing sterol precursors was isolated using the above protocol. Dols from roots were purified using HPLC/UV (fractions eluted at R_t_ 20–33 min). Sterols from roots were isolated from the HPLC/UV eluate by TLC as described above. HPLC/ESI-MS analysis of polyisoprenoids and GC/MS analysis of phytosterols were performed as described earlier [32] with the aid of an Ultra-Performance Liquid 630 Chromatograph ACQUITY UPLC I-Class (Waters Inc.) coupled with a Synapt G2-S HDMS (Waters) mass spectrometer equipped with an electrospray ion source (ESI) and a q-TOF type mass analyzer and Agilent 7890A gas chromatograph coupled to an Agilent 5975C MS Detector under electron impact ionization (70 eV), respectively.

For analysis of labeled pigments they were isolated from lyophilized material [42] and subjected to LC–APCI/MS. Chlorophylls and carotenoids were analyzed by LC–APCI/MS according to the previously described protocol [43] using a high resolution Q-TOF mass spectrometer UHR-MAXIS (Bruker Daltonics) with an UHPLC UltiMate 3000 equipped with a PDA detector (Dionex Softron).

### Estimation of ^2^H enrichment levels and deuteriation patterns of metabolically labeled polyisoprenoids and phytosterols — analysis of MS spectra

Mass spectra of a quality allowing for the calculations of deuterium incorporation (for all variants of metabolic feeding) were obtained only for dominating polyisoprenoids (Pren-11, and Dol-16) and phytosterols (campesterol, stigmasterol, sitosterol). For polyisoprenoid alcohols, signals comprising the isotopic envelope of the most abundant adducts in the mass spectra (sodiated [M + Na]^+^, potassiated [M + K]^+^ or ammoniated [M + NH_4_]^+^) were used for the calculations.

The proposed procedure for analyzing the MS data was aimed at separating the three independent phenomena which contribute to the mass of a given molecule. Thus, it was assumed that the experimentally observed distribution of isotopologues is the convolution of three independent distributions describing the following features:

1. Natural ^13^C abundance is described by the binomial distribution B(n,p^13^C,k), where p^13^C — the natural ^13^C abundance estimated independently from MS spectra recorded for the native products, k — number of ^13^C atoms out of n carbon atoms present in the molecule; *n* = 5·N for polyisoprenoids (N — number of isoprene units in Pren and Dol molecules), *n* = 29 for sitosterol and stigmasterol, and *n* = 28 for campesterol.
2. ^2^H enrichment due to the substrate used is described by the binomial distribution B(d·N,p^2^H,k), where *d* = 2 for ^2^H_2_-DX and 3 for ^2^H_3_-MVL, p^2^H — the relative contribution of unlabeled (i.e. ^1^H) molecules in ^2^H-labeled precursors (estimated independently for both precursors), *k* — number of ^2^H atoms out of d·N locations at which the hydrogen atoms in the molecule can be replaced with ^2^H, N — number of isoprene units.
3. z(i = 0–N) — unknown distribution describing the substrate-dependent cross-talk between the MEP and MVA pathways, where i — number of isoprene units derived from the MEP pathway, while the remaining N-i units are derived from the MVA pathway.

For native (of natural isotopic abundance, i.e. synthesized in plant tissues from photosynthesis-derived precursors) polyisoprenoids, their isotopologue distribution associated with the natural ^13^C abundance was modeled according to the binomial distribution B(n,p^13^C,k). The appropriate model was implemented in Origin Pro (ver. 9.6.0). The natural ^13^C abundance (p^13^C) was estimated as 1.02 ± 0.01% (which is close to the average ^13^C natural abundance of 1.109%) using a global fit applied to the isotopic envelopes determined for Dol-16 and Pren-11.

For each experiment performed with deuteriated precursors, the values of 13 (Pren-11) or 18 (Dol-16) parameters (p^2^H and N + 1 values z(0..N)) were estimated using an appropriate written *ad hoc* function with a standard non-linear fitting procedure (nls) in the R package (R Core Team (2019). R: A language and environment for statistical computing. R Foundation for Statistical Computing, Vienna, Austria. URL https://www.R-project.org/ version 3.6.1).

The obtained data were analyzed regarding either the deuteriation level or the deuteriation pattern. The deuteriation level is the efficiency of deuterium incorporation into a given metabolite. It was calculated as the fraction (%) of the total area of the signals assigned to deuteriated isotopologues relative to the area of all isotopologues, both deuteriated and non-deuteriated, identified in the MS spectrum of a given compound. The deuteriation pattern describes the profile of isotopologues as a function of the number of deuteriated isoprene units in the polyisoprenoid alcohol (*k* = 0–N).

The MS spectra of phytosterols were analyzed using a similar methodology as described above for polyisoprenoids. For phytosterols, signals of molecular ions were taken into account. ^13^C abundance was assumed for both native (synthesized from photosynthesis-derived precursors) and deuteriated molecules of phytosterols of a particular type to be identical with that estimated for Pren-11 and Dol-16.

### The MS-based mechanistic model of polyisoprenoid synthesis

The deuteriation patterns for Pren-11 and Dol-16 were normalized relative to the total population of a given molecule (Supplementary Figure S5). A general power function describing the theoretical distribution resulting from the ‘step-by-step' elongation process (equation 1) was then fitted to a few initial turns of the enzymatic machinery. Note that data for monoisotopic ^12^C Pren-11 or Dol-16, i.e. corresponding to *k* = 0, were excluded from the analysis.

1 $$a+(b-a)\cdot p^{k}\;2or\;a+(b-a)\cdot p^{N-k},$$

for MVA and MEP pathways, respectively

where a and b stand for asymptotic populations of natural isotopic abundance and fully deuteriated isoprene units in a macromolecule, p represents the probability of a subsequent step of polyisoprenoid chain elongation to occur via the assumed pathway, *k* and N–*k* are the total numbers of isoprene units derived from MVA and MEP pathway, respectively, N is the total number of isoprene units in the molecule.

### Statistical analysis

Statistical significance of the effects of specific inhibitors and light conditions was determined by Student's *t* test (*α* = 0.01).

## Results

### Profiles of selected isoprenoids in the leaves and roots of Arabidopsis plants

HPLC/UV analysis of the lipid fraction isolated from leaves of hydroponically grown Arabidopsis plants revealed the presence of a single family of Prens composed of 9, 10, 11 and 12 isoprene units (i.u.) accompanied by a single family of Dols built of 15, 16 and 17 i.u. (Table 1). The roots of these plants contained two families of Dols: a dominating family (Dol-15 to Dol-17) and a less abundant family (Dol-20 to Dol-24), accompanied by traces of the respective Prens. The profile of phytosterols, composed of campesterol, stigmasterol and sitosterol as dominating compounds, was similar for both organs (Table 1). The main plastidial pigments identified in the leaves of hydroponically grown Arabidopsis plants were chlorophylls (a and b) and carotenoids (lutein, β-carotene, neoxanthin and violaxanthin) (Table 1). On the contrary to leaves, the carotenoid content in the roots was very low and only traces of pigments (lutein, β-carotene, violaxanthin, neoxanthin) were detected in this organ (Table 1).

**Table 1:** Profile and content of selected isoprenoids in the leaves and roots of hydroponically grown Arabidopsis

	Leaves		Roots	
	composition	content (µg/g FW)	composition	content (µg/g FW)
**Polyprenols**	Pren-9, **Pren-10**, Pren-11, Pren-12	16.4 ± 3.0	Pren-16, Pren-21	traces
**Dolichols**	Dol-15, **Dol-16**, Dol-17	1.4 ± 0.2	Dol-15, **Dol-16**, Dol-17 Dol-20, **Dol-21**, Dol-22, Dol-23, Dol-24	3.0 ± 0.3
**Phytosterols**	campesterol, stigmasterol, **sitosterol**	1790 ± 160	campesterol, stigmasterol, **sitosterol**	2530 ± 25
**Pigments**	**chla**, chlb lutein, β-carotene, neoxanthin violaxanthin	1400 ± 80 300 ± 30	not detected **lutein**, β-carotene, neoxanthin, violaxanthin	- traces

Compounds dominating within each class are bolded. The content of isoprenoids was estimated in tissues of 5-week old plants grown under short-day (SD) conditions. Presented is the mean ± SD (*n* = 5).

### Inhibitors specific for the MVA and MEP pathways divergently influence the accumulation of polyprenols and dolichols

Next, we estimated the content of polyisoprenoids together with phytosterols and plastidial pigments (considered ‘marker’ products of the MVA and MEP pathways, respectively) in the leaves and roots of inhibitor-treated (Figure 1B) and control age-matched untreated plants. Concentrations of the applied chemicals (100 µM for lovastatin, LOV, and 400 µM for fosmidomycin, FSM) were chosen based on previously described experiments [44].

**Figure 2.**
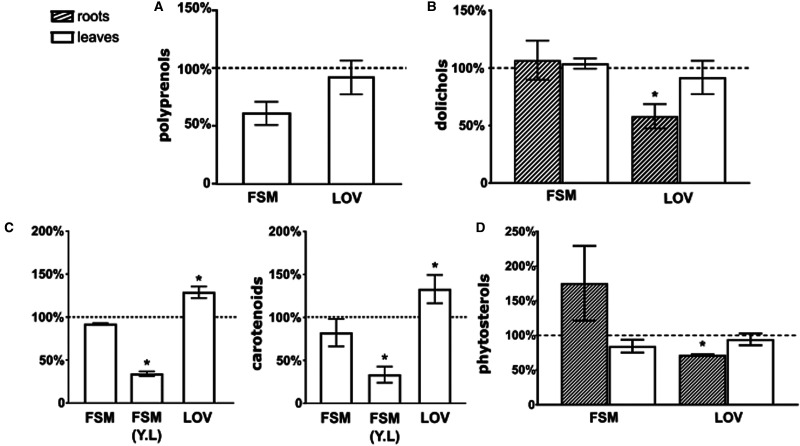
Change of the content of polyisoprenoid alcohols, plastidial pigments and phytosterols in leaves, young leaves (Y.L) and roots of *A. thaliana* treated with specific inhibitors of the MEP and MVA pathways (FSM and LOV, respectively). Plants grown for 5 weeks under SD conditions were treated with inhibitors for 72 h. Values are shown as percentage of the value measured in non-treated plants. Data are mean (±SD) of three independent experiments. *P*-values were estimated by Student's *t*-test, * *P* < 0.05.

Quantitative analysis of the selected isoprenoids revealed inhibitor-induced changes in both tissues. In the roots, LOV treatment resulted in a considerably reduced content of Dols and phytosterols (∼58% and 64% of control, respectively) (Figure 2) while FSM did not affect Dol accumulation but increased that of phytosterols (∼180% of control). The content of pigments was too low for reliable quantification.

In the leaves, LOV treatment resulted in a slight decrease in the content of Dols (∼80% of control) while that of phytosterols remained unchanged (∼98% of control). In contrast with Dols, LOV did not affect the content of Prens (∼95% of control). Surprisingly, LOV induced an increase in the content of pigments (∼125% and 135% of control for chlorophylls and carotenoids, respectively). FSM treatment did not alter the content of Dols while that of phytosterols was slightly decreased (∼86% of control). Most interestingly, FSM caused a considerable decrease in the content of Prens (61% of control) while the level of plastidial pigments was decreased only a little (∼90% of control). Nevertheless, when only young rosette leaves were analyzed, a prominent reduction in pigment content (42% and 32% of control for chlorophylls and carotenoids, respectively) was noted (Figure 2) indicating that FSM mediates perturbations in the metabolic flux toward pigments, at least in young tissues.

The changed levels of the isoprenoid pathway end-products noted both in leaves and in roots fully confirmed that the inhibitors were taken up from the medium by the root system and transported to the leaves. The observed changes of the content of ‘marker’ isoprenoids in response to the inhibitors suggest tight interconnection and reciprocal dynamic regulation of the activity of the MEP and MVA pathways. Furthermore, although the opposing effects of LOV and FSM treatment on Dol and Pren levels suggest divergent mechanisms of their formation, the biosynthetic origin of polyisoprenoids cannot be unambiguously deduced basing on this data.

### Light stimulates accumulation of polyprenols and lowers that of dolichols in Arabidopsis leaves

**Figure 3.**
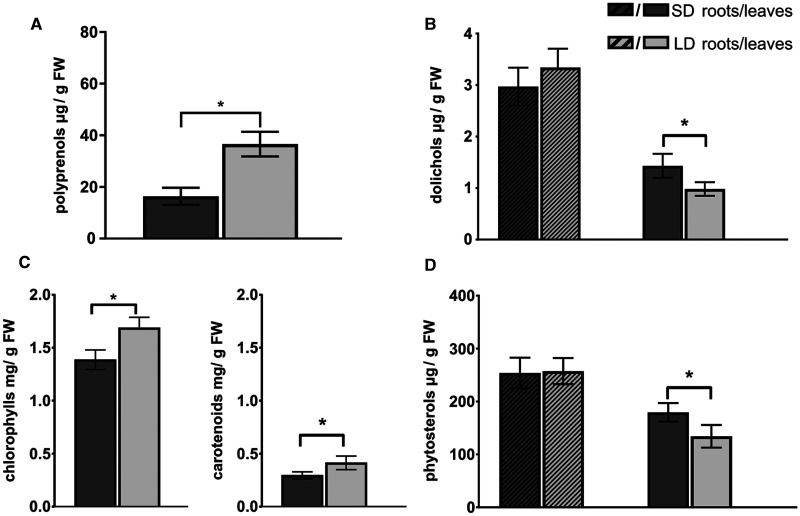
Influence of photoperiod on the content of polyisoprenoid alcohols, plastidial pigments and phytosterols in leaves (empty bars) and roots (hatched bars) of *A. thaliana* plants. Plants were grown under short day (SD, dark gray) or long day (LD, light gray) conditions. Data are mean (±SD) of five independent experiments. *P*-values were estimated by Student's *t*-test, * *P* < 0.05.

To further investigate the involvement of the MVA and MEP pathways in Pren and Dol biosynthesis we were looking for a stimulus that could have contradictory impact on the flux through both pathways toward their end products. Light seemed a good candidate since it down-regulates the MVA pathway and induces the MEP pathway [2,45]. Thus, Arabidopsis plants were cultivated for 4 weeks under short day (SD) or long day (LD) conditions (12 h light or 16 h light, respectively). Additionally, plants grown for 3 weeks under LD conditions were transferred for one more week to continuous light (LD/CL) (Supplementary Table S1). Light exposure did not affect the level of analyzed lipids in the roots but clear changes were observed in the leaves. The highest content of Dols was observed in the leaves of SD grown plants, contrary to Prens for which a statistically significant light-dose dependent increase was noted (2-fold higher for LD and 3-fold higher for LD/CL than SD plants) (Figure 3 and Supplementary Table S1). Phytosterol accumulation followed the same profile as Dols (Figure 3). For chlorophylls and carotenoids an increase was observed for LD plants (comparing to SD plants, Figure 3) and then a subsequent decrease for LD/CL plants (Supplementary Table S1).

The opposite effects of light on the accumulation of plastidial pigments and phytosterols in leaves are in agreement with the influence of light on the MEP and MVA pathway activity (stimulatory and inhibitory, respectively) reported earlier [46,47]. Importantly, the divergent effects of light observed for Prens and Dols further confirm the distinct biosynthetic origin of these two groups of polyisoprenoids.

### Specific precursors of the MVA and MEP pathways are utilized *in planta* for metabolic labeling of isoprenoids

**Figure 4.**
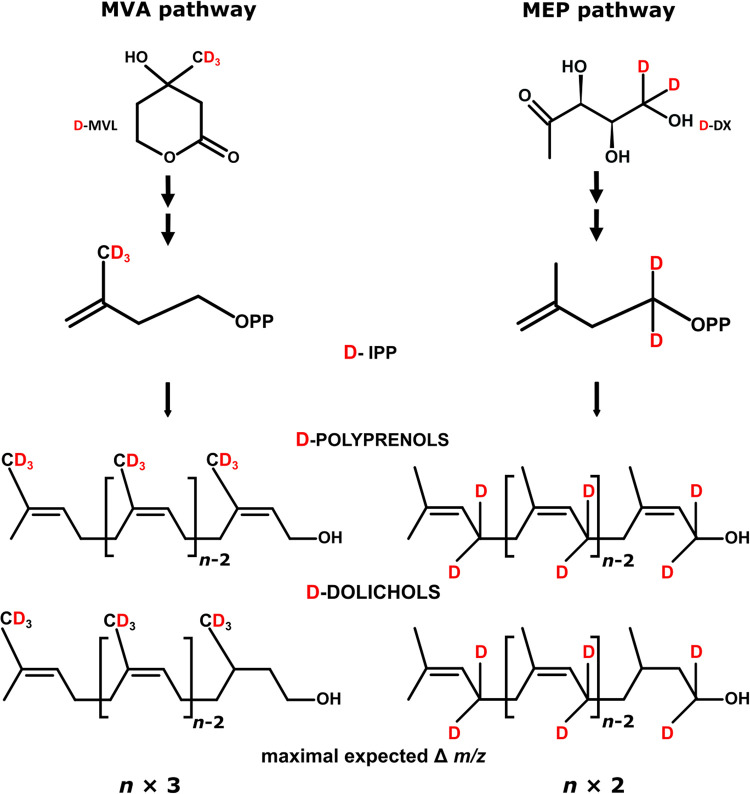
Theoretical labeling patterns. Positions of deuterium atoms in the molecules of the applied deuteriated precursors of the MVA pathway (D-MVL) and MEP pathway (D-DX), the subsequently formed D-IPP (with pathway-specific deuteriation pattern), and the resulting fully deuteriated polyisoprenoid alcohols, presuming their synthesis exclusively via either the MVA (containing three deuterium atoms per isoprene unit) or the MEP pathway (containing two deuterium atoms per isoprene unit). Consequently, maximal expected increase in the *m/z* of polyisoprenoid alcohol is indicated as *Δm/z*. D denotes deuterium atoms, n stands for the number of isoprene units in polyisoprenoid alcohol molecule.

*In planta*, metabolic labeling experiments were designed to address the role of the MVA and MEP pathways in polyisoprenoid biosynthesis in leaves and roots. To diminish metabolic perturbations exerted by exogenous sugars [48] we used deuteriated isotopomers of specific precursors of either the MVA or MEP pathway, respectively, mevalonic acid (MVA) and 1-deoxy-D-xylulose phosphate (DXP), (Figure 1B) which are synthesized in the cell by key enzymes of these routes (HMGR and DXS) [2]. The metabolic labeling experiments described below employed deuteriated precursors administered in their inactive forms, facilitating absorption by the roots in the hydroponic system, i.e. mevalonic acid lactone (D-MVL) and 1-deoxy-D-xylulose (D-DX). According to the current state of knowledge, the conversion of MVL to mevalonic acid (the active form) takes place spontaneously inside the plant cell at physiological pH ([37,38] for more references see [49]) while DX is efficiently phosphorylated by a specific kinase [50] to form DXP. When the specifically labeled compounds (6,6,6-^2^H_3_)MVL (D-MVL) and (5,5-^2^H_2_)1-deoxy-D-xylulose (D-DX) are employed, specifically deuteriated D-IPP and D-DMAPP are formed (Figure 4) and then further incorporated into various isoprenoid backbones via their respective biosynthetic routes. The theoretical labeling patterns of the synthesized isoprenoids can be predicted. Such patterns, assuming the biosynthesis of end products from D-IPP/D-DMAPP derived exclusively from either the MVA or the MEP route, are presented for polyisoprenoids (Figure 4) and for ‘marker’ isoprenoids (Supplementary Figure S1).

**Figure 5.**
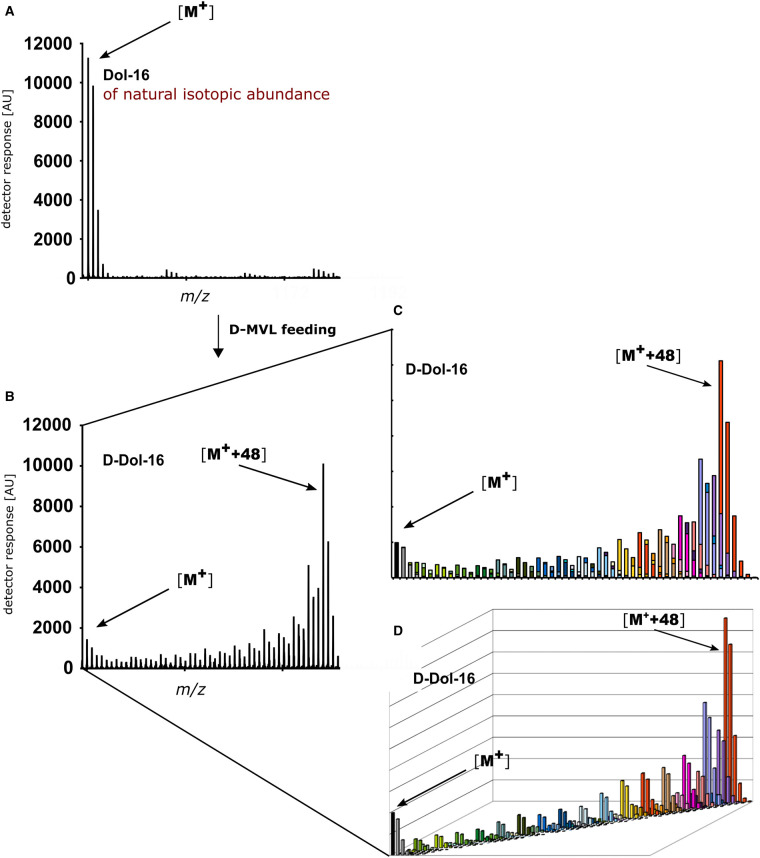
Scheme presenting the procedure of evaluation of isotopic envelopes (composition of isotopologues) of metabolically deuteriated D-Dol-16 isolated from roots of plants fed with D-MVL. (**A**) Mass spectrum of Dol-16 of natural isotopic ^13^C abundance isolated from roots of plants grown in the absence of exogenous precursor; (**B**) mass spectrum of metabolically deuteriated D-Dol-16; (**C** and **D**) deconvoluted mass spectra of metabolically deuteriated D-Dol-16 showing combination of ^13^C natural abundance and ^2^H labeling profiles obtained as a result of mathematical processing of (**B**). It permitted to depict overlapping signals of D-Dol-16 isotopologues with various number of D atoms but the same *m/z* value (**C**) and individual ^13^C isotopic envelope of each deuteriated D-Dol-16 isotopologue (**D**, diagonal axis). Diversely colored bars were used to indicate deuteriated isotopologues containing specific number of D atoms; unified color code is used in panels (**C**) and (**D**); non-deuteriated isotopologues of Dol-16 molecular ions [M^+^] (black and grey bars) and D-Dol-16 isotopologues with maximal number of deuterium atoms anticipated after feeding with D-MVL [M^+^ + 48] (burgundy colored bars) are indicated by arrows.

Two sets of labeling experiments were performed. During a ‘single precursor’ labeling experiment plants were grown in the presence of a single labeled precursor (D-DX or D-MVL). During a ‘competitive’ labeling experiment plants were simultaneously fed with both precursors (one deuteriated and one with natural isotopic abundance; depending on the type of precursor that is deuteriated, we denote these experiments either D-DX/MVL or D-MVL/DX). This second type of experiment was introduced in order to minimize the perturbations of metabolic fluxes caused by feeding with a single precursor. In a single-precursor labeling experiment deuteriated IPP/DMAPP are shuffled with natural isotopic abundance counterparts stemming from photosynthesis, while in a competitive labeling experiment they are additionally shuffled with a pool of exogenous unlabeled precursors. Consequently, the mass spectra of the labeled isoprenoid compounds comprise complex sets of signals derived both from natural isotopic abundance (Figure 5A) and from variously deuteriated (Figure 5B) molecules, as shown for Dol-16.

Moreover, we assumed that an isotopic envelope of a particular isoprenoid compound is in fact a convolution of three isotopic distributions (see Materials and Methods), i.e. two binomial distributions associated with (i) the natural abundance of ^13^C (∼1.1%) and (ii) deuterium abundance in the applied substrate (see below), and (iii) the unknown distribution of labeled isoprene units resulting from cross-talk between the MEP and MVA pathways. Every deuterium-enriched molecule is supposed to replicate the ^13^C isotopic envelope of the compound of natural isotopic abundance (Figure 5C). As a result, signals corresponding to differentially deuteriated molecules would overlap showing the same *m/z* values (e.g. an *m/z* M + 4 signal is generated by molecules containing four atoms of deuterium and 0 atoms of ^13^C or three atoms of deuterium and 1 of ^13^C, etc.) (Figure 5D). Consequently, molecules containing the same number of deuterium atoms contribute to signals of different *m/z* due to different number of ^13^C atoms (Figure 5D).

Therefore, to quantify deuterium incorporation the raw mass spectra of metabolically deuteriated isoprenoids were numerically processed to dissect the effects of the metabolic feeding with deuteriated precursors from that resulting from natural ^13^C abundance (∼1.1%). The outcome is described below for the analyzed groups of isoprenoids. We present the results in terms of ‘deuteriation level’ and ‘deuteriation pattern’.

**Figure 6.**
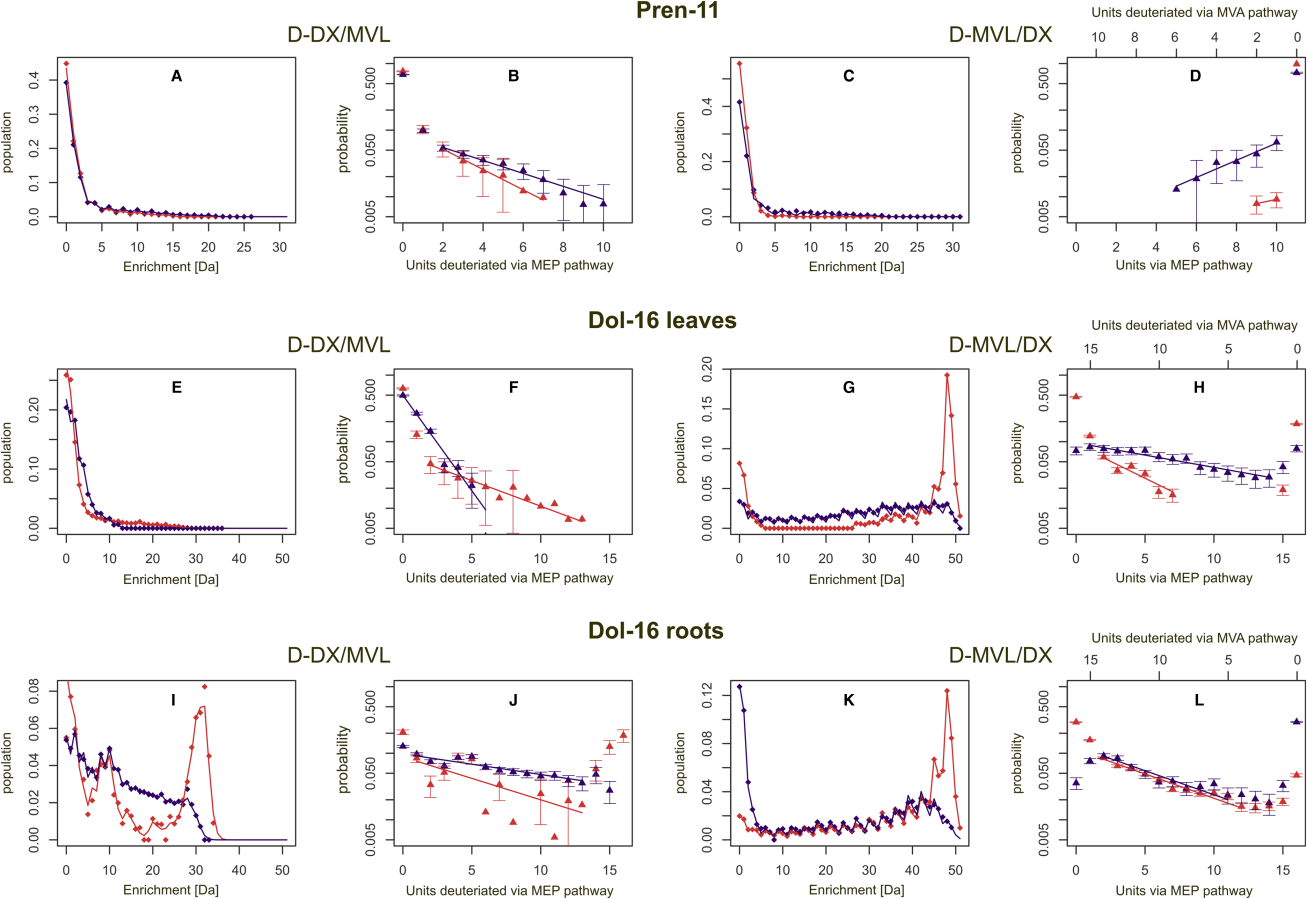
Labeling of polyisoprenoids in *Arabidopsis* tissues using D-DX or D-MVL as precursors — comparison of the results of competitive vs. single-precursor experiments. Diamonds depict the experimentally recorded mass spectra of the indicated polyisoprenoid compounds and illustrate the populations of isotopologues showing particular isotopic enrichment (Da) while triangles show the number of deuteriated isoprene units, i.e. the pattern of deuteriation, estimated for the indicated polyisoprenoid compound. Please note that the deuteriation pattern is presented as a contribution of the MEP pathway and is shown in the logarithmic scale. Additionally, for Pren-11 the deuteriation pattern is presented also as a contribution of the MVA pathway (the scale is displayed above the panels). Shown are overlaid data obtained after feeding of plants with the indicated precursors, either D-DX or D-MVL, used either as single precursors (red symbols and lines) or in combination with the appropriate natural isotopic abundance precursors (blue symbols and lines). The traces of the mass spectra were arbitrarily normalized to 100% (A, C, E, G, I, K). Values presenting the deuteriation pattern (i.e. the distributions of deuteriated isoprene units) are means ± SD (B,D,F,H,J,L). Please note that the maximal expected isotopic enrichment of Pren-n/Dol-n after D-DX or D-MVL feeding is, respectively, (M) + 2n or (M) + 3n, see also Figure 4. Each row presents data for the indicated polyisoprenoid compound, i.e. Pren-11 (**A**–**D**) or Dol-16 (**E**–**H**) isolated from leaves or Dol-16 isolated from roots (**I**–**L**).

- Deuteriation level of the compound of interest corresponds to the fraction (%) of deuteriated isotopologues possessing at least one labeled isoprene unit and is used as an indicator of the efficiency of incorporation of deuterium (summarized in Table 2).
- Deuteriation pattern of a polyisoprenoid alcohol (Pren-11 or Dol-16) presents the distribution of isotopologues with an indicated number of deuteriated isoprene units (*k* = 0..N, see section Methods) (summarized in Figure 6, even columns).

It should be kept in mind that due to the low natural isotopic abundance of deuterium (∼0.01%) its effect on the isotopic envelopes of the analyzed isoprenoids is negligible.

**Table 2:** Quantitative analysis of deuterium incorporation into polyisoprenoid molecules

	Labeling with D-DX						Labeling with D-MVL					
	D-DX			D-DX/MVL			D-MVL			D-MVL/DX		
	Isotopologues (%)		Average number of deuteriated i.u	Isotopologues (%)		Average number of deuteriated i.u	Isotopologues (%)		Average number of deuteriated i.u	Isotopologues (%)		Average number of deuteriated i.u
Prenologue tissue	Non-deuteriated	Fully deuteriated		Non-deuteriated	Fully deuteriated		Non-deuteriated	Fully deuteriated		Non-deuteriated	Fully deuteriated	
Pren-11 leaves	76 ± 1	0 ± 2	0.7	68 ± 1	0 ± 1	1.1	98 ± 1	0 ± 2	<0.1	73 ± 2	0 ± 2	0.6
Dol-16 leaves	63 ± 1	0 ± 2	1.5	50 ± 1	0 ± 1	1.0	19 ± 1	47 ± 1	11.9	8 ± 1	7 ± 1	8.7
Dol-16 roots	21 ± 2	19 ± 4	7.9	13 ± 1	0 ± 1	5.8	5 ± 1	29 ± 1	11.8	29 ± 1	4 ± 1	7.1

### Metabolic labeling of polyisoprenoids

Supplementation of Arabidopsis growth medium with exogenous D-MVL or D-DX resulted in the appearance of additional signals in the mass spectra of the analyzed polyisoprenoids. It indicated the incorporation of labeled precursors (raw MS spectra are shown in Supplementary Figure S2 and Supplementary Figure S3). Deconvolution of these spectra led to identification of the distribution of deuteriated isotopologues for Pren-11 (Supplementary Figure S2) and Dol-16 (Supplementary Figure S3). Moreover, the ^2^H enrichment of the deuteriated precursors was evaluated by fitting an appropriate model to the experimental distributions of Pren-11 and Dol-16 isotopologues upon labeling with a single precursor. The level of non-deuteriated precursors was estimated at 2.9 ± 0.9% for ^2^H_2_-DX and 0.7 ± 0.1% for ^2^H_3_-MVL, which corresponds to the enrichment of 97.1 ± 0.9% for ^2^H_2_-DX and 99.3 ± 0.1% for ^2^H_3_-MVL.

### Pren-11

Analysis of the labeling of Pren-11 was performed exclusively in the leaves (Figure 6A,C for D-DX and D-MVL, respectively).

#### Deuteriation level of Pren-11

The estimated deuteriation level of Pren-11 was ∼23% for feeding with D-DX and 1.7% for feeding with D-MVL, and it was increased upon both competitive labeling conditions, reaching ∼32% for D-DX/MVL and 25% for D-MVL/DX (Table 2). However, no fully labeled Pren-11 molecules were detected in any labeling conditions (Figure 6A,C).

Furthermore, the incorporation of deuterium from D-DX was more efficient when plants fed with D-DX for 4 weeks were further transferred for a short-time to fresh medium containing D-DX (reaching ∼34% after 24 h of additional labeling and 46% after 48 h, Supplementary Figure S4). This data suggests that the low efficiency of Pren deuteriation upon feeding with D-DX might be caused, among others, by a high rate of Pren degradation. The turnover of either Prens or Dols, however, has not been studied in plant tissues.

#### Deuteriation pattern of Pren-11

For D-DX feeding, the maximal number of MEP-derived isoprene units in Pren-11 molecules (Figure 6B) was ∼7 i.u. for labeling exclusively with D-DX, and 10 i.u. for D-DX/MVL labeling. For D-MVL labeling, the maximal number of MVA-derived i.u. was ∼2 for sole D-MVL, and 6 i.u. for the D-MVL/DX experiment (Figure 6D, see also section Numerical analysis of mass spectra of polyisoprenoids). These data show that the MEP pathway predominates over the MVA pathway as the source of IPP utilized for Pren-11 synthesis.

### Dol-16

The labeling of Dol-16 was analyzed separately in the leaves and roots (Figure 6, middle and lower row, respectively).

#### Deuteriation level of Dol-16

##### In leaves

Deuterium incorporation turned out to be higher for labeling with D-MVL than with D-DX (deuteriation levels ∼81% and 37%, respectively, Table 2). While fully labeled Dol-16 isotopologues were predominating in D-MVL labeling experiments (∼47%), no such molecules were detected for D-DX labeling (Figure 6E,G). Under competitive labeling conditions, deuterium incorporation was increased compared with single-precursor feeding (Table 2): the deuteriation level was ∼92% for D-MVL/DX experiments (with a decrease in the population of fully labeled isotopologues to ∼7%) and 50% for D-DX/MVL (here the population of partially labeled isotopologues was increased, at the expense of unlabeled molecules) (Figure 6E,G).

##### In roots

Dol-16 was efficiently labeled after feeding with either D-MVL or D-DX (95% and 79% deuteriation levels, respectively) (Table 2). Signals corresponding to fully labeled Dol-16 were detected in both of these experiments, although they were far less abundant for D-DX than for D-MVL labeling (respectively 19% and 29%). Compared with single-precursor feeding, in the competitive experiments deuterium incorporation was considerably decreased for D-MVL/DX (deuteriation level 71%) but increased for the D-DX/MVL experiment (deuteriation level 87%) (Table 2). Also a clear decrease in the population of fully labeled isotopologues was observed for both competitive experiments (Figure 6I,K) indicating a ‘mosaic’ origin of Dol, at least in roots. Moreover, an increase in the pool of unlabeled isotopologues (reaching 29%) was noted for the D-MVL/DX experiment.

#### Deuteriation pattern of Dol-16

Analysis of MS spectra allowed us to estimate the number of MEP-derived isoprene units in Dol-16 molecules (Figure 6).

##### In leaves

When D-DX was used as a single precursor, Dol-16 labeling reached 13 i.u. while the D-DX/MVL competitive experiment resulted in labeling of 4–5 i.u. (Figure 6F). Upon application of D-MVL alone at least 9 i.u. were formed via the MVA pathway (i.e. up to seven derived from the MEP pathway) while upon D-MVL/DX feeding all possible deuteriated isotopologues were observed (from 0 to 16 i.u. labeled, with all remaining units derived from the MEP pathway) (Figure 6H).

##### In roots

Labeling with D-DX, used either as a single precursor or in combination with MVL (D-DX/MVL), revealed that all 16 i.u. were labeled, i.e. derived from the MEP pathway, although the population of isotopologues containing high numbers (*n* > 6) of deuteriated i.u. was decreased upon competitive labeling conditions. Moreover, we observed a local maximum at 8–10 Da for D-DX used as a single precursor which might suggest that preferably ∼4–5 i.u. are derived from the MEP pathway (Figure 6J). For D-MVL and D-MVL/DX labeling, Dol-16 molecules with all isoprene units labeled could be identified and the population of isotopologues containing high numbers of deuteriated isoprene units (*n* > 13) was much larger for the D-MVL/DX experiment than for the D-DX/MVL experiment (Figure 6L).

In summary, the estimated average numbers of deuteriated isoprene units (Table 2) reveal that in the leaves D-DX incorporation into both Pren-11 and Dol-16 was marginal. This indicates that a locally synthesized photosynthesis-derived natural isotopic abundance DX pool probably dilutes the exogenously administered D-DX. The effect of D-MVL application was different for Pren than for Dol: for Dol-16 molecules the labeling was strong, while for Pren-11 the incorporation of this precursor was very low. These results suggest that in the leaves the metabolic origin of Pren-11 and Dol-16 — and consequently also the site of their synthesis — is different.

In the roots both precursors, D-MVL and D-DX, are efficiently incorporated into Dol-16 molecules. However, the average number of deuteriated i.u. per molecule (Table 2) indicates a preference of the Dol biosynthetic route for MVA-derived precursors over MEP-derived units.

### Numerical analysis of mass spectra of polyisoprenoids

Subsequent numerical analysis of the labeling data revealed that the distribution of deuteriated isoprene units followed an exponential trend as presented in Figure 6B,D,F,H,J,L (values on the Y axis are shown in the logarithmic scale, for comparison see Supplementary Figure S5 where a linear scale is used). The decreasing log-linear trends of the labeling pattern depicted in these panels indicate that the synthesis of a polyisoprenoid molecule, except for the case of Pren-11 labeling with D-MVL, is initiated using a MEP-derived isoprene unit and the probability of each subsequent isoprene unit of the chain to be deuteriated and supplied via the MEP pathway, (*P*, equal to the exponent of the slope) remains constant independently of the type of precursor used and the labeling conditions employed (Supplementary Table S2). Similarly, a constant probability of the elongation of a Dol chain has previously been noted in yeast [51]. Since the probability of elongation depends on the balance of locally available substrates, its values are different in leaves and roots. Interestingly, the increasing log-linear trends of the labeling pattern observed upon labeling of Pren-11 with D-MVL (*P* > 1), which suggests that the probability of elongation exceeds 1, are apparently illogical and indicate that the tested model is not applicable for Pren-11 upon D-MVL labeling; it also suggests that access of the MVA-derived precursors to the site of Pren-11 biosynthesis remains limited which again confirms the predominance of the MEP pathway in Pren-11 synthesis. It should be kept in mind however that due to the limited deuteriation level observed for Pren-11 (e.g. 25% for D-MVL/DX experiment) and resulting data noise these results require careful interpretation. In addition during D-MVL/DX competitive labeling experiments when both precursors are administered to plants the conversion of MVL to IPP used to synthesize might not follow the classical isoprenoid pathway (for comments see Supplementary Figure S2).

It is worth noting that in the competitive labeling experiments (except for Dol-16 labeling with D-DX/MVL in the leaves) the presence of exogenous MVL or DX of natural isotopic abundance increases the contribution to the final product of exogenous deuteriated D-DX or D-MVL, respectively.

Taken together the application of specific precursors suggests that Pren-11 is favorably derived from MEP pathway precursors, while Dol-16 is derived — both in roots and in leaves — concomitantly from MEP and MVA pathway precursors with a consistently stronger contribution of the MVA pathway (∼13–12 i.u., versus 3–4 i.u. from the MEP pathway).

### Modeling of MS spectra of polyisoprenoids

To get a more in-depth understanding of the mechanism of polyisoprenoid biosynthesis, modeling of their MS spectra was employed. This analysis, described in detail in the Supplemental Material (Supplementary Figure S5), shows the differences between the biosynthesis of Pren-11 and Dol-16. The model describes the process of polyisoprenoid chain synthesis: the chain is initially synthesized by elongation with isoprene units derived from one of the two pathways, either MEP or MVA, and further elongated with units derived from the other pathway. Interestingly, the estimated pathway balance differs qualitatively depending on the type of analyzed product (Pren-11 or Dol-16) and, in the latter case, differs quantitatively depending on tissue type (roots or leaves).

### Pren-11

The model shows that Pren-11 is preferentially synthesized from endogenous MEP precursors derived from photosynthesis. At the same time, the presence of exogenous DX activates an additional minor route that leads to Pren molecules of a ‘mosaic’ character. Thus, some molecules consist of units derived from exogenous MEP precursors and endogenous precursors (Supplementary Figure S5A,B) with a minor contribution of MVA-derived precursors.

### Dol-16 in leaves

The model indicates that upon precursor feeding the Dol-16 produced in the leaves is of mixed origin, and it can be expected that the same is valid under native conditions. The model suggests that the enzymatic machinery responsible for the formation of Dol can use both exogenous and endogenous substrates (Supplementary Figure S5C and Figure 5D). When applied to D-MVL labeling, the model shows the existence of two pools of Dol-16 molecules. A pool derived predominantly from the endogenous precursors of the MEP pathway accompanies another pool derived from both (MEP- and MVA-originating) types of exogenous and endogenous precursors and/or their metabolites. The latter is evidenced by a substantial disagreement between the estimated value of the intercept and the experimentally observed level of Dol-16 of natural isotopic abundance. Finally, the estimated MEP-pathway origin of 4–5 isoprene units in the Dol-16 molecule is indicated by a systematic deviation from the model (Supplementary Figure S5D). Such an effect is in line with our previous data for Dols isolated from the hairy roots after metabolic labeling with (^13^C)glucose suggesting that the initial isoprene units of Dol molecules are of MEP-pathway origin [31]. So far, the biosynthetic origin of Dol in the leaf has not been analyzed. However, this observation suggests that a similar pathway leads to Dol in the leaf and root.

### Dol-16 in roots

Dol-16 in roots, similarly to that in leaves, is formed with the contribution of both the MEP and MVA pathways. However, contrary to the situation in the leaves, in the roots, another pool of Dol-16 molecules originating solely from the MEP pathway can be detected (Figure 6I,J vs. Figure 6E,F). The existence of such two pools of Dol-16 molecules — one derived only from endogenous precursors of the MEP pathway and the other derived simultaneously from exogenous and endogenous precursors (and/or their metabolites) of both pathways — is supported by all four labeling experiments. Furthermore, again 3–5 isoprene units in each Dol-16 molecule originate favorably from the MEP pathway (Supplementary Figure S5E), and such effect is much stronger in the roots than in the leaves. As noted above, the results of *in vivo* labeling of Dol-16 in roots with pathway-specific precursors are in line with our data for Dols isolated from the hairy roots after labeling with (^13^C)glucose.

### Metabolic labeling of phytosterols

Labeled molecules of the analyzed phytosterol species (campesterol, stigmasterol, sitosterol) were detected both in the leaves and in the roots of plants grown in media supplemented with either type of deuteriated isoprenoid precursor (Supplementary Table S3 and Supplementary Figure S6).

#### In roots

In single-precursor labeling experiments, phytosterols were more efficiently labeled with D-MVL (the population of deuteriated compounds was ∼50% and 70% for sitosterol and stigmasterol, respectively, and only ∼30% for campesterol) than with D-DX (∼12–15% for all analyzed phytosterols). Still, these results indicate that the MEP pathway could, to some extent, contribute to the biosynthesis of phytosterols under the tested conditions. Not only did the labeling levels of individual phytosterols differ, but dissimilar deuteriation profiles were observed for stigmasterol and sitosterol in most of the labeling experiments. This observation is, at first glance, surprising since stigmasterol is synthesized from sitosterol through C-22 desaturation [52]. It should be, however, kept in mind that labeling analysis by mass spectrometry does not differentiate different pools of a metabolite, potentially derived from different carbon sources involved in their biosynthesis. Stigmasterol might be thus derived from a sitosterol pool different from the bulk pool.

In the competitive labeling experiments, the presence of MVL of natural isotopic abundance (D-DX/MVL labeling) increased the incorporation of deuterium (∼28–38% of molecules deuteriated). In contrast, the addition of natural isotopic abundance DX (D-MVL/DX labeling) caused a decrease in the deuteriation for sitosterol and stigmasterol (∼31% and 40%, respectively) and an increase for campesterol (∼45%).

#### In leaves

The incorporation of D-MVL into phytosterols was low (∼10–20%) and so was the incorporation of D-DX (∼20%). In the competitive labeling experiments the presence of natural isotopic abundance DX (D-MVL/DX experiment) substantially increased the incorporation of deuterium (∼70% for campesterol, 74% for stigmasterol, and 58% for sitosterol), similarly as was the case for polyisoprenoid alcohols. The presence of MVL of natural isotopic abundance (D-DX/MVL experiment) slightly decreased the deuteriation of campesterol and sitosterol (∼10%) but increased that of stigmasterol (∼35%).

Among all tested conditions the highest level of deuterium incorporation was found for D-MVL/DX competitive labeling in the leaves. The relatively low level of D-MVL incorporation prompted us to analyze the profiles of phytosterols and intermediates of the phytosterol biosynthetic pathway. For plants grown in media supplemented with exogenous MVL, both labeled or unlabeled, the amount of these intermediates was considerably elevated compared with control plants, e.g. after D-DX/MVL labeling we observed a 64- and 12-fold increase in 24-methylene cycloartanol levels in leaves and roots, respectively (Supplementary Figure S7). At the same time, the end-product level (the analyzed phytosterols) was not changed in these samples compared with control plants or plants fed with DX.

The calculated distribution of deuteriated isotopologues was not in full accordance with the pattern predicted for labeling via the classical (cycloartenol-mediated) phytosterol biosynthetic route (Supplementary Figure S1) i.e. the presence of M + 10 Da isotopologues for all analyzed leaf phytosterols theoretically unattainable using D-MVL. However, it should be kept in mind that alternative phytosterol biosynthetic pathways have been described too. Thus, *in vitro* studies of Arabidopsis sterol methyltransferase 2 (SMT2) revealed its potential to form end products with M + 10 Da [53]. Additionally, D-MVL and D-MVL/DX labeling experiments led to the formation of M + 12 isotopologues (for campesterol in leaves and sitosterol in roots, respectively), thus exceeding the maximal number of incorporated deuterium atoms predicted for the classical phytosterol biosynthetic pathway via cycloartenol. Still, it should be kept in mind that M + 12 isotopologues are expected for phytosterols formed via lanosterol instead of cycloartenol. The lanosterol pathway has been shown to be operational in Arabidopsis [54]. Taken together, the formation of isotopologues corresponding to M + 10 and M + 12 is fully justified; hence recorded deuteriation pattern suggests that feeding with exogenous specific precursors may induce flux through alternative branches in phytosterol biosynthesis and formation of non-classical phytosterol intermediates.

### Metabolic labeling of plastidial pigments

Analysis of labeling was performed exclusively for pigments isolated from leaves. Although the presence of carotenoids (lutein and β-carotene) was confirmed in the roots of hydroponically grown Arabidopsis plants by HPLC/UV (data not shown), their content in the samples obtained from metabolically labeled plants was too low to permit mass spectrometry analysis. Molecular ions, convenient for the analysis of deuterium incorporation, were detected in LC/APCI mass spectra of lutein and β-carotene (Supplementary Figure S8 and Supplementary Figure S9). However, reliable quantitative analysis of deuterium incorporation levels was not possible because of technical impediments (unspecific and inconsistent fragmentation, unidentified contaminants overlapping with the signals of expected deuteriated carotenoids), evidenced especially in the spectra of β-carotene. In the mass spectra of lutein isolated from plants fed with D-DX or D-MVL, we could observe additional signals of *m/z* higher than those found in samples from control plants. This shows successful albeit low incorporation of labeled precursors of both pathways. A qualitative assessment of these spectra suggests that feeding with D-DX was more efficient for carotenoid labeling than D-DX/MVL feeding, which resulted in the lowest labeling efficiency of all tested variants. The incorporation of D-MVL into carotenoids was marginal in both tested variants (D-MVL and D-MVL/DX). A qualitative assessment of these spectra suggests that feeding with D-DX was more efficient for carotenoid labeling than D-DX/MVL feeding. The incorporation of D-MVL into carotenoids was very low in both tested variants (D-MVL and D-MVL/DX).

## Discussion

The contributions of the MVA and MEP pathways to Pren synthesis have never been documented. In contrast, the biosynthetic origin of Dols has so far been investigated only in hairy root cultures, indicating the involvement of both pathways [31,32]. Thus, intact plants were employed as the experimental model in this work to study the biosynthesis of both groups of polyisoprenoid alcohols simultaneously and to investigate possible differences in the routes of Dol biosynthesis in leaves and roots.

The first approach we took was to evaluate changes in the accumulation of dolichols and Prens upon differentiated metabolic flow through the pathways induced by photoperiod or by the application of inhibitors. These experiments indicated that the biosynthetic origin of Prens and Dols is divergent. The accumulation of Prens in long-day conditions (together with chlorophylls and carotenoids), as well as the decrease in their content after application of fosmidomycin clearly show that they are derived from the MEP pathway, while changes in Dol content revealed a pattern similar to that noted for phytosterols, connecting these compounds with the MVA pathway. Divergent regulation of the MVA and MEP pathways by light is well documented [45]. Light stimulates the transcription of several MEP-pathway genes in Arabidopsis [55,56] but down-regulates the expression of MVA-pathway genes and concomitantly sterol levels [57,58]. A positive correlation between the levels of plastidial pigments and other MEP-derived isoprenoids with the day length has been previously reported for soil-grown plants [59–61]. Furthermore, light-driven accumulation of Prens has been observed in numerous plant species during the vegetative season [62].

However, inhibitor-triggered changes in the accumulation of phytosterols and plastidial pigments differed from those anticipated for ‘marker’ compounds. For phytosterols, the changes were organ-dependent and in the leaves differed from those expected for MVA-end products. The significant inhibitory effect of lovastatin treatment towards phytosterols in the roots but not in the leaves might reflect different biosynthesis rates in these organs. This supposition is supported by a previous study, which showed that in *Nicotiana benthamiana* leaves, the activity of the key MVA pathway enzymes HMGR (HMG-CoA reductase) and HMGS (HMG-CoA synthase) was substantially lower than in the roots, and it was further decreasing upon plant senescence [63]. Furthermore, the substantial decrease in the content of plastidial pigments observed upon fosmidomycin treatment in young leaves but only modest in mature rosette leaves might be explained by developmental regulation. Importantly, the ‘marker’ isoprenoids were affected upon blockage of the other pathway. Interestingly, we observed compensatory modulation of the MVA or MEP pathway productivity which might implicate a cross-flow of intermediates between compartments upon blockage of either route; such an effect has been reported previously in Arabidopsis seedlings [44]. It should be mentioned that both inhibitors were shown to alter the expression of multiple genes outside the isoprenoid biosynthetic routes [44]. Thus their effects might be pleiotropic, e.g. by disruption of protein prenylation [64,65]. Additionally, side effects caused by their toxicity cannot be excluded. In line with this, it has been suggested in the literature [2,44] that conclusions regarding the biosynthetic origin of the studied isoprenoid compounds must not rely on data obtained using inhibitors but must be complemented with other experimental approaches.

**Figure 7.**
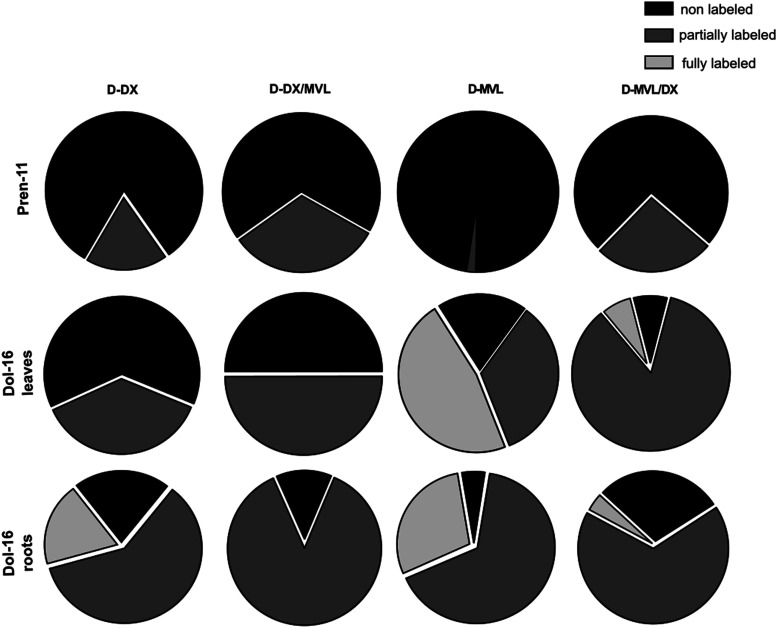
Relative abundance of isotopologues of deuteriated Pren-11 and Dol-16 after metabolic feeding with specific deuteriated precursors. Fractions of unlabeled, partially labeled and fully labeled molecules were calculated for D-Pren-11 from leaves and D-Dol-16 isolated from leaves or roots after single-precursor and competitive labeling experiments.

Our second approach, metabolic labeling with specific precursors of the pathways, was designed to produce quantitative data, and it held the potential to reveal even low inputs from the analyzed pathways. It should be kept in mind, however, that although the application of the specific precursors is considered not to be *per se* toxic to plants unless used in high concentrations, it still may alter the intermediate exchange rate between the MVA and MEP [30]. Moreover, incorporating of DX into plastidial metabolites requires its phosphorylation in the cytoplasm [50] and subsequent import into the plastid. The obtained results confirmed that the biosynthetic origin of Dols and Prens is different. In line with previous experiments, Prens preferentially incorporated MEP-derived precursors. Only a minute fraction of MVL-labeled Pren-11 molecules was detected, suggesting some adaptive capacity of the Pren biosynthetic machinery. The analysis of labeled Dols indicated contributions from both pathways to Dol synthesis *in planta*. Although at first glance, the ‘mosaic' character of Dols seems not fully consistent with the results of the inhibitor studies, the observed inconsistencies might be the effect of fluctuations resulting from the metabolic cross-talk between the MVA and MEP pathways throughout plant growth and development. The results might also reflect a potential for pathway cross-flow, which is attained due to the increased availability of the specific precursors. Interestingly, either of the pathways alone seems sufficient to provide the IPP needed for Dol chain biosynthesis. However, overall labeling was more efficient after supplementation with the deuteriated precursor of the MVA pathway, both in leaves and roots (Figure 7 and Table 2).

In contrast with the generally accepted opinion that phytosterols are MVA pathway products, metabolic labeling also revealed some MEP pathway contribution to their formation, again indicating pathway cross-talk. In line with this, substantial cross-talk between pathways has been recently observed in the *in planta* model for ‘marker’ isoprenoids (phytosterols, carotenoids) after feeding young (two-week old) cotton seedlings with labeled specific precursors. Interestingly, the analysis of labeled isoprenoids indicated a more intensive flux of labeled MVA into the specific MEP end products than in the opposite direction. Additionally the labeling efficiency was tissue-dependent [30]. Numerous other studies employing metabolic labeling revealed that for the formation of various isoprenoids plants often use isoprenoid-producing pathways against their classical assignments, e.g. MEP contributes to sesquiterpenes or phytosterols while MVA — to carotenoids, α-tocopherol, plastoquinone, for details see excellent review papers [2,66]. Furthermore, some plant species (e.g. strawberry) possess a multifunctional enzyme catalyzing the formation of both mono- and sesquiterpenes; in ripe strawberry fruit it is localized to the cytosol and not, as expected, to the plastids. Accordingly, the exact product formed depends on the availability of suitable substrates [67].

Moreover, elucidations of the mechanisms regulating the cross-talk led to interesting conclusions reported in the literature. Hence upon treatment of Arabidopsis seedlings with pathway-specific inhibitors a cross-talk between MEP and MVA pathway was documented at the metabolite level, but lack of correlation between gene expression patterns indicated the role of posttranscriptional processes in regulating flux through isoprenoid metabolic pathways [44]. Subsequent studies on Arabidopsis mutants revealed that LOVASTATIN INSENSITIVE 1 (*LOI1*) and CHLOROPLAST BIOGENESIS6 (*CLB6*) genes control the posttranscriptional regulation of the MEP and MVA pathways [68,69]. Collectively, posttranscriptional and posttranslational regulation of enzyme levels and activity is responsible for fine-tuning of the MEP and MVA metabolic flux [70]. Moreover, it should be kept in mind that cross-talk level depends on various factors such as plant species, type of tissue, stress conditions which indicates the complex organization of plant isoprenoid metabolism [69,71].

In our study incorporation of labeled precursors into chlorophylls and carotenoids in mature leaves was generally very low. This might suggest that the developmental stage of the plant, with its particular biochemical demands, might define the potential for cross-talk between pathways and thus the labeling outcome. Another important factor affecting the labeling results might be the duration of the feeding with specific precursors due to the divergent turnover rate of isoprenoids. Chlorophylls and carotenoids were shown to undergo constant turnover in mature Arabidopsis leaves even in non-stress conditions [72], which may contribute to their weak deuteriation levels in our experimental conditions. Similar turnover dynamics may apply to plastid-located Prens since ROS quenching is among their postulated biological functions [24].

It is worth noting that polyisoprenoids appear to be attractive model compounds for labeling studies aimed at the elucidation of isoprenoid biosynthesis. Formation of the polyisoprenoid chain directly from IPP minimalizes the possibility of miscalculating label incorporation due to the presence of enzymatic regulatory steps and the accumulation of labeled intermediates as was observed for phytosterols.

To counteract the possible metabolic disturbances triggered by fueling up of one of the pathways through the application of a single precursor (for references see [49]) we came up with the concept of competitive labeling, which involves the concomitant application of both precursors, i.e. a deuteriated precursor of one of the pathways together with the natural isotopic abundance precursor of the other. In general, if both pathways were engaged in the biosynthesis of a particular compound, the fraction of unlabeled or partially labeled molecules of this compound would increase, at the expense of fully labeled molecules, due to isotopic dilution. Indeed, such an effect was observed for Dol-16, further supporting the ‘mosaic’ character of Dols (Figure 7). Furthermore, modeling of mass spectra performed here revealed, both for roots and leaves, that Dol-16 molecules are preferably synthesized from 3–4 isoprene units from the MEP pathway and 12–13 units from the MVA pathway. Our previous labeling experiments, performed in Arabidopsis hairy root cultures, led to the conclusion that as many as 10–11 i.u. were of MEP origin [32]. This discrepancy most probably reflects the differences between both experimental models, i.e. hairy root cultures vs. intact plants. Anyhow, data shown here obtained for labeling with pathway-specific precursors alike those recorded for labeling with glucose [32] document contribution of both, the MEP and MVA, pathways to biosynthesis of Dols. Future studies employing transcriptomic approaches and correlation of these data with metabolomics are needed to present the comprehensive landscape of biosynthetic routes leading to polyisoprenoids and showing flow of metabolic intermediates together with the effects exerted by exogenous precursors.

Finally, the intriguing conclusion from analysis of labeling data that under the conditions of the competitive labeling experiments two subpopulations of differentially labeled Pren-11 and Dol-16 were detected deserves special attention. It might suggest that differentially labeled Pren molecules are formed in the two functionally or perhaps even spatially separated putative plastidial sub-compartments comprising the plastidial isoprenoid-generating pathway:

- (i) one uses predominantly endogenous precursors and has very limited access to exogenous DX and/or its metabolites and is simultaneously blocked for exogenous MVL-derived metabolites,
- (ii) the other one uses both exogenous and endogenous precursors and/or their metabolites and receives influx from both pathways.

Any of the methods commonly used to analyze the cross-talk of the MEP and MVA pathway, whether the genetic or chemical blockade of the pathway or supplementation with pathway specific precursors affects the homeostasis of the plant cell and perturb the metabolic flow. It should be kept in mind however, that in contrast with the former methods which lead to visible phenotypic changes/lethality [2] feeding with DX and MVA, used either as a single precursor or combination of both, appeared well-tolerated by plant and permitted to estimate the relative contribution of both pathways to polyisoprenoids.

The methodology used in this report to analyze polyisoprenoid biosynthetic pathway assumes that exogenous precursors, DX and MVL, are accessible to the synthetic machinery of both isoprenoid generating systems. Indeed, the results obtained here and manifold studies using metabolic labeling, confirm this assumption. Still, the possible effect of metabolite channeling, which might control the influx of precursors, cannot be neglected and should be addressed in future projects. It is an open question whether polyisoprenoid biosynthetic route comprises a metabolon — ‘supramolecular complex of sequential metabolic enzymes and cellular structural elements' [73] or rather a transient enzyme–enzyme assembly which could mediate metabolite channeling. Although channeling of metabolites has been evidenced for e.g. TCA cycle, glycolysis, and biosynthesis of purine nucleotides [74], still the concept of metabolon, and terpenosome/isoprenolosome in particular, requires experimental proof. Our labeling studies might suggest the existence of such functionally/spatially separated enzyme–enzyme assemblies, linked to the glycolysis performing enzymes, easily consuming deoxyxylulose phosphate as a metabolite formed directly from the products of glycolysis. While consumption of the exogenously supplied DX, which undergoes phosphorylation in the cytoplasm, might require *ad hoc* formation of the complex using these DXP molecules to form isoprenoids.

### *cis*-Prenyltransferase and the cross-talk of the MEP and MVA pathways

**Figure 8.**
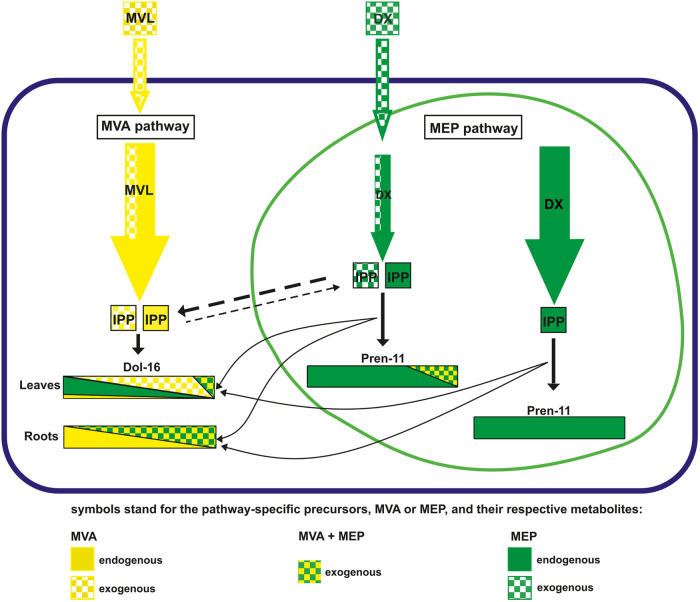
Spatial organization of polyisoprenoid biosynthesis in the plant cell — effect of exogenous precursors. Polyprenols (Prens) are formed and accumulated in plastids while the formation of dolichols (Dols) is initiated in the plastids but finalized in the cytoplasm. Putative export of plastidial oligoprenyl precursors to the cytoplasm required for biosynthesis of Dol is indicated (solid arrows). The putative bidirectional exchange of intermediates between the plastids and the cytoplasm, which includes isopentenyl diphosphate (IPP, dashed arrows), is marked too. The exogenously supplied pathway-specific precursors, mevalonolactone (MVL, yellow-checkered square) and deoxyxylulose (DX, green-checkered square) enter their respective pathways and get metabolized to IPP (checkered squares) in parallel with their endogenous counterparts (filled squares). Interestingly, exogenous DX seems to induce a plastidial MEP subroute which is capable of using both exogenous and endogenous DX and operates in parallel to the main flow of the MEP pathway which utilizes solely endogenous DX. Consequently, two fractions of Prens are synthesized in plastids: one is synthesized solely from IPP originating from endogenous DX (filled green rectangle) and the other one (less abundant) is synthesized from IPP derived from both endo- and exogenous DX and/or MVL (green-white, yellow-white, green-yellow checkered rectangles). Both pools of oligoprenyl/polyprenyl precursors are subsequently used to form Dols in the cytoplasm. Although exogenous MVL and DX contribute significantly to Dol biosynthesis both in roots and leaves, their relative contribution in these organs is different — to illustrate this observation rectangles standing for Pren and Dol are split into sections proportionally (see Figure 6 for comparison) to the observed incorporation of the indicated endogenous and exogenous precursors.

Since our previous reports suggested that the synthesis of Dols is initiated in plastids and finalized in the cytoplasm [31], the model presented here might suggest that cytoplasmic *cis*-prenyltransferase(s), CPT(s), sequentially elongating the growing polyisoprenoid chain, has independent access to these two pools of differentially labeled oligoprenyl precursors. Such a model of spatial regulation of Dol biosynthesis could be accomplished by spatially- and/or temporally -regulated export of oligoprenyl precursors from plastids towards cytoplasmic CPT(s) via yet unknown transporters (Figure 8). Although no mechanism of such translocation is known yet, it might engage stromules (long protrusions that bud from plastids localized in the proximity of the endoplasmic reticulum) as is the case for the export of geraniol upon the synthesis of indole alkaloids in *Catharantus roseus* [75]; the export of oligoprenyl intermediates into the close environment of CPT(s) should facilitate the final steps of Dol biosynthesis. Indeed, stromules have already been identified in several plant species, including Arabidopsis [76].

An alternative explanation of how Dol is synthesized might come from the suggested ability of cytoplasmic/ER enzymes to access the pool of non-polar plastidial metabolites, most likely through plastid:ER membrane interaction domains [77]. This transorganellar access mechanism, described for tocopherol and carotenoid synthesis, might also appear valid for Dol synthesis. According to this scenario, plastidial oligoprenyl precursors are accessible to CPTs localized at the cytoplasm/ER interface. They also undergo elongation to form Prens, which subsequently serve as substrates for ER-associated polyprenol reductase (PPRD2) [11]. This implies that CPT7 [19] and CPT3 [12] function independently to form Prens and Dol in Arabidopsis, similarly to the role played by two distinct CPTs in spinach leaves [78]. Such a tentative model satisfactorily explains that upon D-DX feeding, Dol-16 isotopologues in leaves are built either solely of unlabeled or labeled isoprene units. All other scenarios would involve the synthesis of Pren-16 inside plastids by CPT7 (the only CPT so far ascribed to plastids). Since CPT7 is known to catalyze the formation of a family of Prens with Pren-10 and -11 dominating [19], this seems highly improbable. The intriguing concept that Dol synthesis takes place at the ER/cytoplasm interface, using MEP pathway precursors accessible to ER-localized CPT and PPRD2, requires further studies.

## Conclusions

We documented for the first time that in intact plants, the biosynthetic routes leading to Prens and Dols differ in terms of MEP- and MVA-pathway contributions. We show that Prens are derived mainly from the MEP pathway; however, marginal contributions from the MVA pathway are observed under specific conditions. In contrast, Dols are ‘mosaic’ isoprenoids, and the relative input of the MVA and MEP pathways to their synthesis is modulated by the current productivity of these routes.

The model of Dol biosynthesis built on these data is in line with our previous observations noted for hairy root cultures [31,32]. The novel statistical methodology we applied here for labeling results allowed us to elucidate this mechanism in photosynthetically active plants producing unlabeled precursors. Based on the results of the competitive labeling experiments, we propose that DX and MVL and/or their metabolites might re-orchestrate the cellular metabolism of isoprenoids.

The incorporation of deuterium from specific deuteriated precursors, DX or MVL, documents their effective uptake from the medium and transport towards the cellular compartments capable of converting them to IPP, i.e. the plastids and the cytoplasm/ER, respectively. The data presented here show that a limited bi-directional exchange of intermediates between the cytoplasm and the plastids is plausible, as suggested earlier [79,80]; the rate of such a translocation might be regulated by the current metabolic output of the MVA and MEP pathways.

The competitive labeling approach, presented here for the first time, provides additional information on the cross-talk between the MVA and MEP pathways.

## Data Availability

The data needed to evaluate this work are all included in the manuscript, and are available upon request from J.P. (jarek@ibb.waw.pl) and E.S. (ewas@ibb.waw.pl).

## Abbreviations

Dol: dolichol
DX: deoxyxylulose
FSM: fosmidomycin sodium salt
HPLC/ESI-MS: high performance liquid chromatography coupled with electrospray ionization mass spectrometry detection
LOV: lovastatin
MEP: methylerythritol phosphate pathway
MVA: mevalonate pathway
MVL: mevalonolactone
Pren: polyprenol
