# Supplementary material for: Divergent contribution of the MVA and MEP pathways to the formation of polyprenols and dolichols in Arabidopsis
Source: Biochem J. 2023 Apr 21;480(8):495–520. doi: 10.1042/BCJ20220578 (PMC10212524; doi:10.1042/BCJ20220578)
Supplement: Supplementary Material [file BCJ-480-495-s1.pdf]

Supplementary Information

Divergent contribution of the MVA and MEP pathways to the formation of polyprenols and dolichols in Arabidopsis

Agata Lipko[1,*,#], Cezary Pączkowski[2], Laura Perez-Fons[3], Paul D. Fraser[3], Magdalena Kania[4], Marta Hoffman-Sommer[1], Witold Danikiewicz[4], Michel Rohmer[5], Jaroslaw Poznanski[1,*], Ewa Swiezewska[1,*]

[1]Institute of Biochemistry and Biophysics Polish Academy of Sciences, Warsaw, Poland
[2]Department of Plant Biochemistry, Faculty of Biology, University of Warsaw, Poland
[3]School of Biological Sciences, Royal Holloway, University of London, Egham Hill, UK
[4]Institute of Organic Chemistry, Polish Academy of Sciences, Warsaw, Poland
[5]Université de Strasbourg/CNRS, Institut Le Bel, Strasbourg, France

[#]Present address: The Nalecz Institute of Biocybernetics and Biomedical Engineering, Laboratory of Nanohybrid Biosystems Regulation Engineering, Polish Academy of Sciences, Warsaw, Poland

List of contents:

Supplementary Tables: S1, S2
Supplementary Figures: S1-S8

Supplementary Table S1. Content of polyisoprenoid alcohols, phytosterols and plastidial pigments in *A. thaliana* plants grown under different light conditions - short day (SD), long day (LD) and continuous light (CL). For CL treatment LD plants were transferred to 24h-light for one week (LD/CL). Data are mean (± SD) of five independent experiments. P-values were obtained by Student's *t*-test, * P < 0.05 (SD vs LD and LD vs LD/CL).

		**Leaves**			**Roots**		
		SD	LD	LD/CL	SD	LD	LD/CL
Polyprenols (µg/g FW)		16±3	37±4*	59±9*	-	-	-
Dolichols (µg/g FW)		1.4±0.2	1.0±0.1*	0.9±0.1	2.97±0.3	3.3±0.3	3.0±0.1
Phytosterols (µg/g FW)		180±16	134±20*	129±15	254±24	257±20	263±26
Pigments (mg/g FW)	chlorophylls	1.4±0.1	1.7±0.1*	1.1±0.1*	-	-	-
	carotenoids	0.3±0.1	0.4±0.1	0.3±0.1	-	-	-

Supplementary Table S2. Metabolic labeling of polyisoprenoids using exogenous precursors – the log-linear trends of the labeling pattern are illustrated by the estimated values (p) of the probability of each subsequent isoprenoid unit to be deuteriated and supplied *via* the MEP pathway (see Figure 6 for comparison).

Polyisoprenoid alcohol	tissue	Exogenous precursor(s) used			
		solely D-DX	D-DX/MVL	solely D-MVL	D-MVL/DX
		p			
Pren-11	leaves	0.70 (0.02)	0.80[a] (0.01)	1.16[b,c]	1.35[a,c](0.04)
Dol-16	leaves	0.84 (0.01)	0.52 (0.02)	0.79 (0.04)	0.92[a] (0.01)
Dol-16	roots	0.86 (0.05)	0.93[a] (0.01)	0.84 (0.01)	0.85[a] (0.02)

a) the presence of exogenous MVL or DX of the natural isotopic abundance increases the contribution of exogenous deuteriated D-DX or D-MVL
b) standard error is not calculated since only two Pren-11 isotopologues of mixed origin were detected
c) p>1 indicates restricted contribution of exogenous MVA to Pren-11 biosynthesis

Supplementary Table S3. Metabolic labeling of phytosterols using exogenous precursors

	labeling with D-DX				labeling with D-MVL			
	D-DX		D-DX/MVL		D-MVL		D-MVL/DX	
	leaves	roots	leaves	roots	leaves	roots	leaves	roots
phytosterol	deuteriation level of isotopologues (%)		deuteriation level of isotopologues (%)		deuteriation level of isotopologues (%)		deuteriation level of isotopologues (%)	
campesterol	20	12	10	34	20	30	70	45
stigmasterol	20	15	34	40	6	70	74	40
sitosterol	20	12	15	30	10	50	58	30

Supplementary Figure S1. Positions of deuterium atoms in the molecules of the deuteriated precursors, (6,6,6(methyl)-$^2H_3$)MVL and (5,5-$^2H_2$)DX, the subsequently formed IPP, and the analyzed phytosterols and carotenoids, presuming their synthesis exclusively via either the MVA or the MEP pathway. D denotes deuterium atoms.

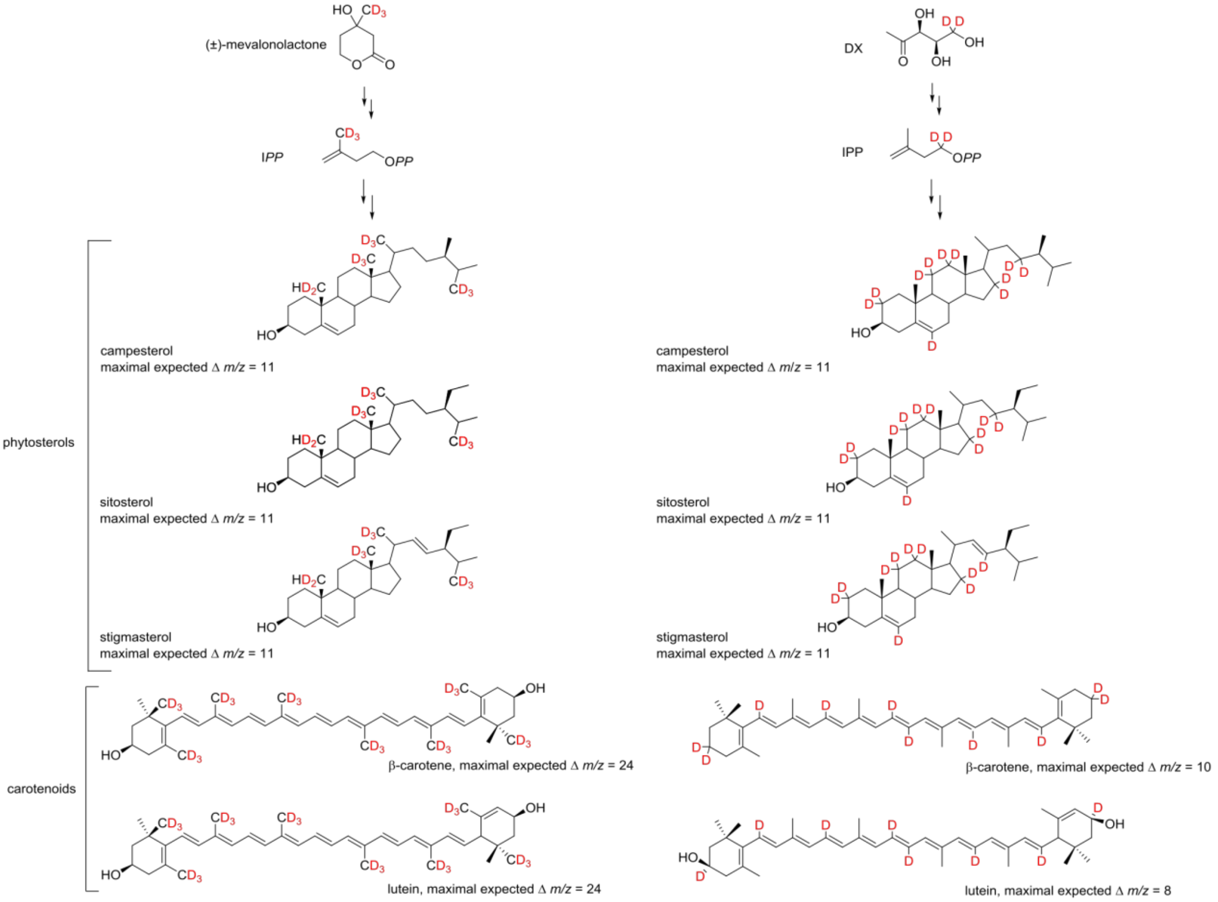

Supplementary Figure S2. Results of metabolic labeling of Pren-11. Shown are data for D-DX (left column) and D-MVL (right column) labeling experiments, data for single precursor (blue bars) vs. competitive (green bars) labeling experiments are juxtaposed.
Upper row: experimentally recorded distribution of integrated raw mass spectra of the deuterium-labeled Pren-11.
Middle row: deuteriation profiles of Pren-11, i.e. deconvoluted raw mass spectra.
Lower row: deuteriation patterns of Pren-11, i.e. distribution of isotopologues with an indicated number of deuteriated isoprene units.

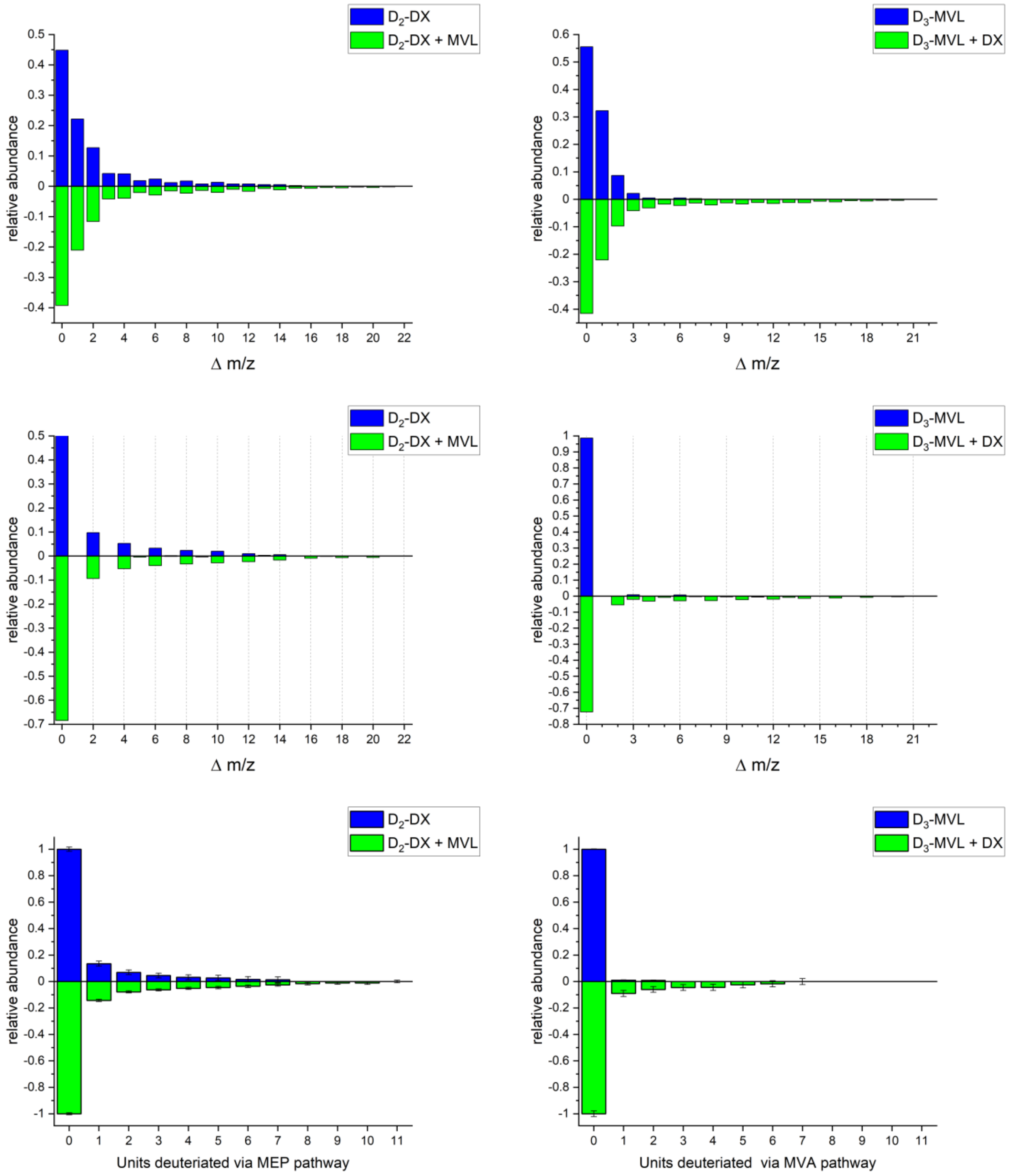

Original mass spectra of Pren-11 recorded for indicated labeling experiment were subjected to numerical processing. Firstly, the procedure of deconvolution (for details see Figure 5) resulted in the deuteriation profile of Pren-11,. which was corrected for natural ^{13}C abundance separately

for each labeling experiment. Secondly, numerical modeling (see Materials and methods) led to estimation of the deutariation pattern of Pren-11 specific for each labeling experiment.

Commentary note to Supplementary Figure S2

Incorporation of precursors used in this study (D-DX or D-MVL) into the molecules of the end-products should result in a precursor-specific distribution of deuterium atoms (Figure 4); consequently, in the mass spectra of polyisoprenoid alcohols one can observe a clear predominance of signals corresponding to every third isotopologue (*m/z* M + 3i) after D-MVL or every second isotopologue (*m/z* M + 2i) after D-DX supplementation, where i=1,...,n and n stands for the number of i.u. in the analyzed molecule. Complex deuteriation profile observed for Pren-11 upon D-MVL/DX labeling might indicate that upon this particular conditions the so called ’mevalonate shunt’ identified in mammals [1] and in insect cells [2] as well as in plants [3] might play some role.

Thus, although the competitive labeling experiments, used here for the first time, provide additional data compared to classical single-precursor labeling, the results must be critically analyzed in the context of a complex regulatory network since flux through the MVA pathway is tightly regulated, mainly at the post-transcriptional and post-translational level [4].

Supplementary Figure S3. Results of metabolic labeling of Dol-16 isolated from leaves (A) and roots (B). Shown are data for D-DX (left column) and D-MVL (right column) labeling experiments, data for single precursor (blue bars) vs. competitive (green bars) labeling experiments are juxtaposed.

For both panels (A) and (B):

Upper row: experimentally recorded distribution of integrated raw mass spectra of the deuterium-labeled Dol-16.

Middle row: deuteriation profiles of Dol-16, i.e. deconvoluted raw mass spectra.

Lower row: deuteriation patterns of Dol-16, i.e. distribution of isotopologues with an indicated number of deuteriated isoprene units.

(A)

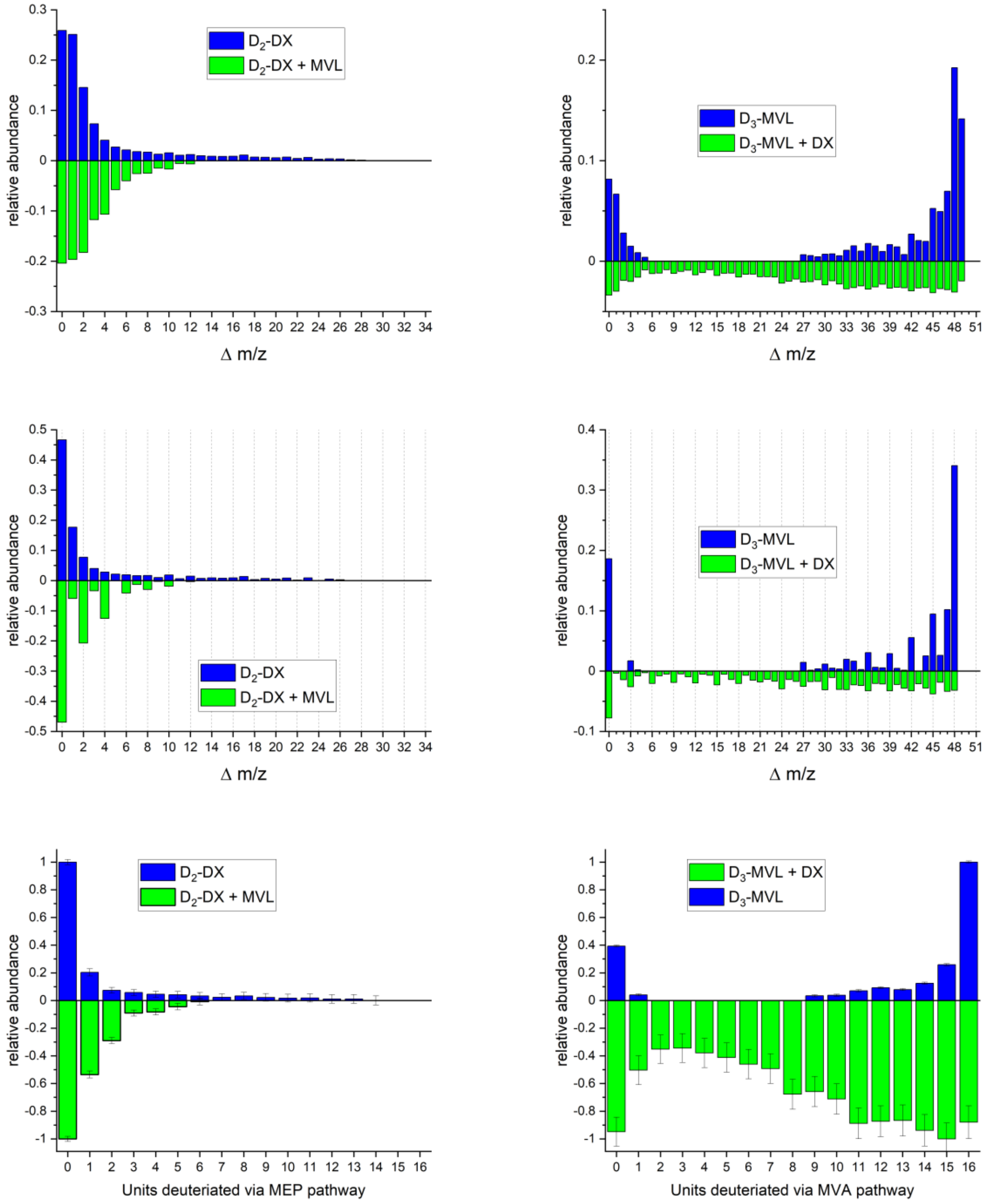

(B)

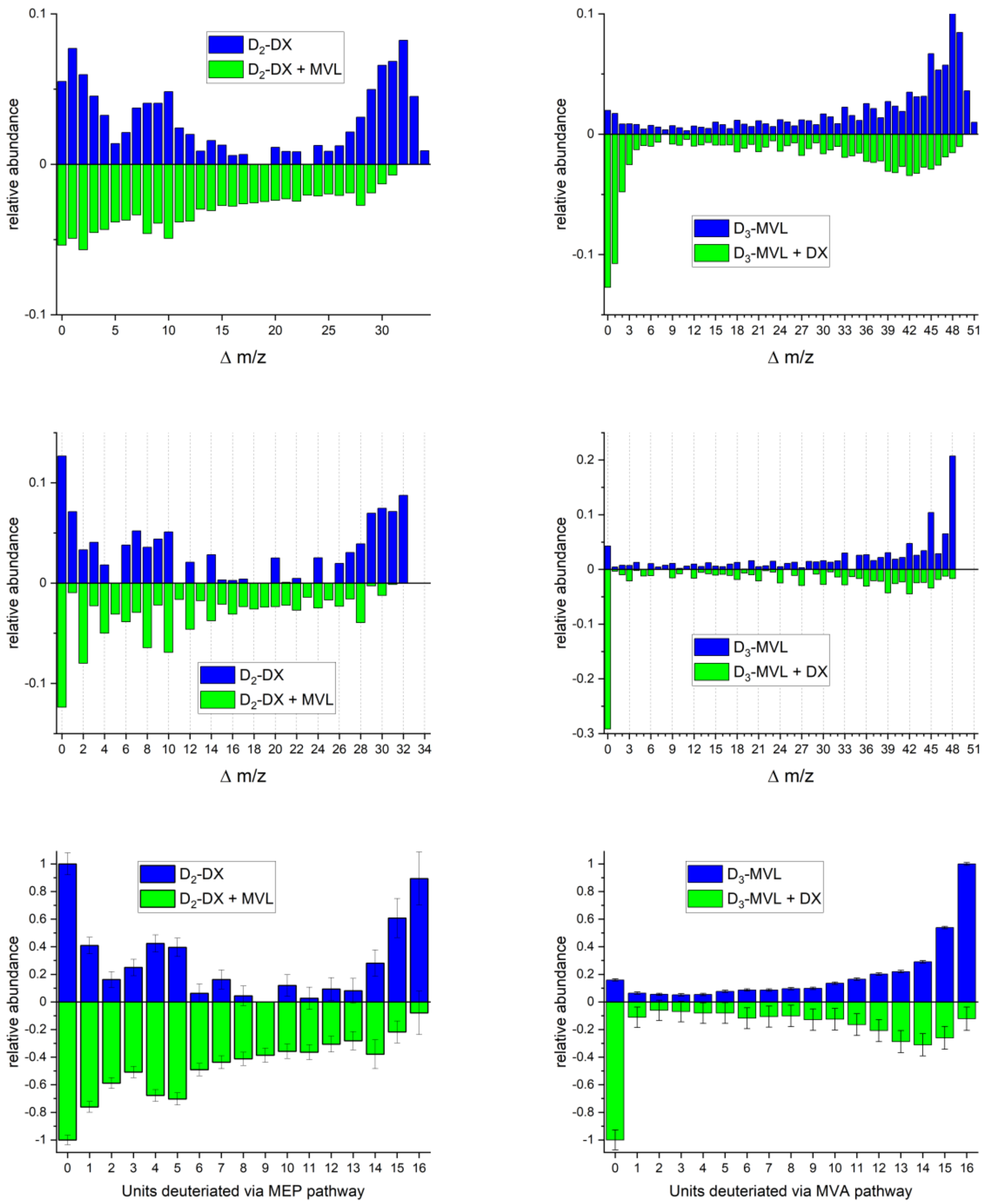

D$_2$-DX
D$_2$-DX + MVL
D$_3$-MVL
D$_3$-MVL + DX
relative abundance
Δ m/z
Units deuteriated via MEP pathway
Units deuteriated via MVA pathway

Supplementary Figure S4. Deuteriation profile of Pren-11 using D-DX as substrate – comparison of various labeling conditions. Arabidopsis plants were grown for 5 weeks in medium containing D-DX (0.5 mM), leaves were harvested and Pren-11 was analyzed using HPLC/ESI-MS. In a parallel experiment plants were grown for 4 weeks in medium with D-DX (0.5 mM), then they were transferred to fresh medium containing the same concentration of D-DX (0.5 mM) for an additional 24 or 48 h (D-DX+24h, D-DX+48h, respectively), and then harvested and analyzed as above. The scale on the Y-axis was modified to better visualize signals of low intensity.

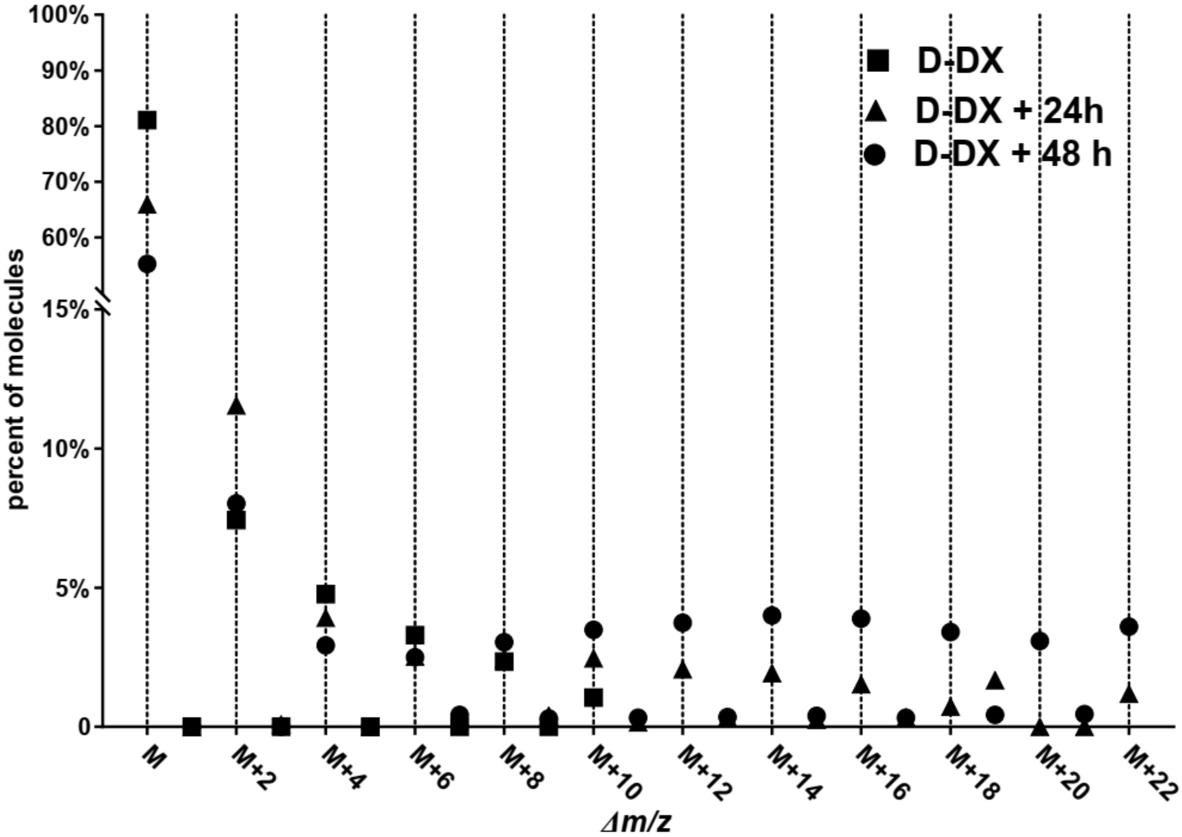

The calculated deuteriation levels (approx. 20%) for the initial labeling period (4 weeks) was increased upon D-DX supplementation (to approx. 30% and 45% for 24 and 48h, respectively).

Supplementary Figure S5. **Labeling of polyisoprenoids in Arabidopsis tissues using D-DX or D-MVL in single-precursor or competitive labeling experiments – the distribution of deuteriated isoprene units (calculated as the contribution of the appropriate pathway) was compared for both types of experiments.**
A and B, Pren-11 isolated from leaves; C and D, Dol-16 isolated from leaves; E and F, Dol-16 isolated from roots. Results of labeling with D-DX and D-MVL are presented in panel A, C, E and B, D, F, respectively.
Shown are overlaid normalized (normalization performed relative to the population of natural isotopic abundance Pren-11 or Dol-16 molecules, respectively, n = 0) distributions of labeled isoprene units deduced from experimental data (mean ± SD) and from the model (solid lines) with 5% confidence limits (red shadows).
To simplify the interpretation the triangle markers are rotated for each series individually - deuteriation derived from D-DX is depicted with vertically-oriented triangles and that derived from D-MVL with horizontally-oriented triangles. Red symbols and lines show data for the single-precursor experiments, blue symbols and lines show data for the competitive labeling experiments, and orange symbols indicate data excluded from the analysis. Please note that data in this figure are presented using the linear scale in contrast to Figure 6 where the logarithmic scale was used.
For details of the results of MS spectra modeling please refer to Commentary Notes to Supplementary Figure S5).

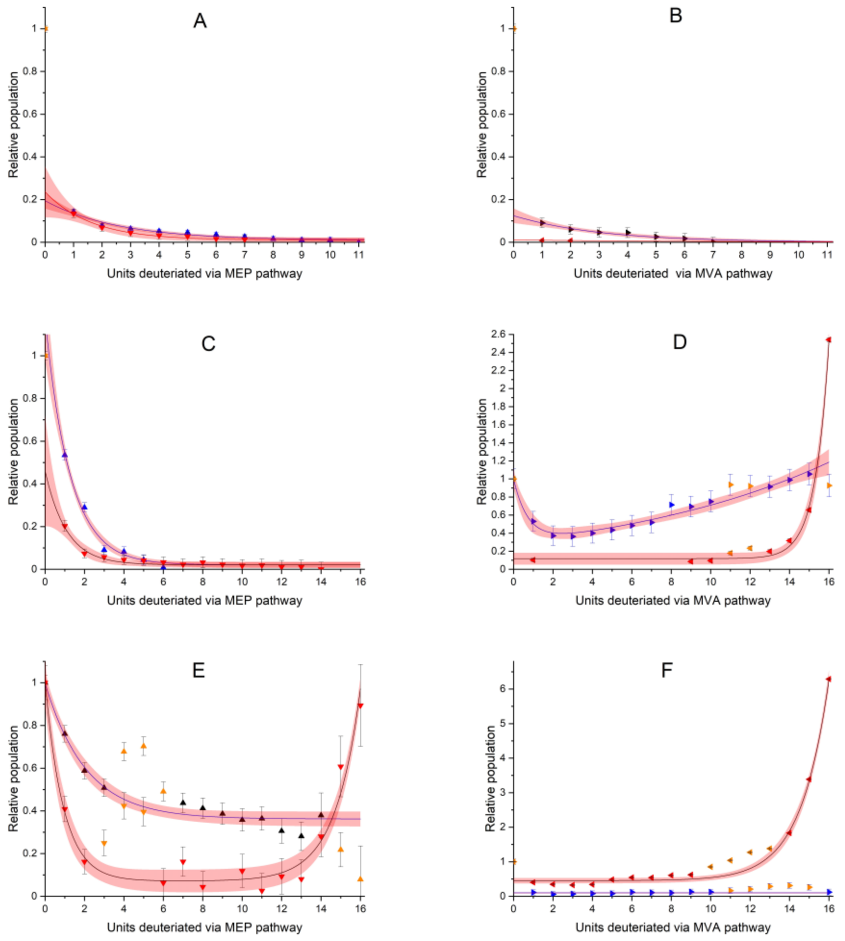

Commentary notes to Supplementary Figure S5

The modeling of MS spectra clearly documents striking differences between the biosynthesis mechanisms for Pren-11 and Dol-16.

The model assumes cooperation of the MEP and MVA pathways during polyisoprenoid formation: the polyisoprenoid chain is synthesized initially from isoprene units derived from one of the two pathways, and then elongated with those produced by the other one.

Pren-11**:** Despite the feeding scheme, a vast majority of Pren-11 molecules were not labeled with ^{2}H. The observed distribution of ^{2}H-labeled isoprene units in Pren-11 is in agreement with the postulated formulae (equation 1, Supplementary Figure S5A,B) for all types of feeding schemes, but the parameters derived from the model for different feeding experiments differ slightly. Since values on the X axis correspond to the number of isoprene units deuteriated *via* the indicated particular pathway, extrapolation to n=0 reflects the native conditions where no exogenous precursors have been used. Thus, extrapolation to n=0 explains only approx. 20% of the experimentally observed population of Pren-11 of the natural isotopic abundance (23 ± 5 or 19 ± 2% for D-DX or D-DX/MVL, respectively, Supplementary Figure S5A). These numbers indicate that approximately 80% of natural isotopic abundance Pren-11 molecules are synthesized in a different way, e.g. their synthesis might proceed in a chloroplast subcompartment not accessible to IPP molecules derived from exogenous D-DX. On the one hand natural isotopic abundance IPP (or some other isoprenoid precursor molecules of the natural isotopic abundance) derived from photosynthesis and originating from the MEP pathway, which are not labeled with ^{2}H, do not mix with those derived from exogenous D-DX and consequently only Pren-11 of the natural isotopic abundance is synthesized. On the other hand the pool of IPP derived from D-DX does mix with unlabeled molecules originating (putatively) from the MEP pathway and as a result a stochastic spectrum of variously labeled Pren-11 molecules is observed. This suggests that the deuteriated D-Pren-11 and natural isotopic abundance Pren-11 molecules are formed in spatially separated plastidial subcompartments, one of which contains biosynthetic machinery capable of using both exogenous D-DX and natural isotopic abundance DX simultaneously while the other is only capable of using native, endogenous substrates.

As expected, only unlabeled Pren-11 molecules (>99%) are observed upon supplementation with solely D-MVL, while, paradoxically, a substantial population of deuteriated Pren-11 is observed when both D-MVL and DX are present in the feeding medium (Supplementary Figure S5B). Both these findings support our hypothesis, established originally for Dol biosynthesis (29), that the synthesis of polyisoprenoid chains – in this case Pren-11 – must be initiated with a MEP-derived IPP molecule and is then continued with IPP originating from the MVA pathway.

Altogether, a consistent model of Pren synthesis emerging from this modeling is as follows: in the absence of exogenous DX the synthesis proceeds using only natural isotopic abundance IPP derived from photosynthesis in chloroplasts, while supplementation with exogenous DX (natural isotopic abundance or deuteriated) somehow activates the MEP pathway in the plastidial subcompartment in which IPP derived from both exogenous precursors (i.e. MVL and DX, both labeled and natural isotopic abundance) is accessible, and as a result Pren-11 of mosaic origin may be synthesized. Still, this fraction represents only 10-20% of the total population of Pren-11 molecules. Summarizing, Pren-11 is preferentially synthesized from photosynthesis-derived precursors, and the activation of an additional minor route that leads to Pren molecules of mosaic MEP/MVA origin takes place only in the presence of exogenous DX.

Dol-16 in the leaves: The distribution of ^{2}H-labeled isoprene units in Dol-16 molecules observed upon supplementation with exogenous D-DX (Supplementary Figure S5C) follows the trend observed already for Pren-11 under similar conditions, though the population of Dol-16 of the natural isotopic abundance containing isoprene units originating from the MEP pathway is visibly higher than that estimated for Pren-11 (Supplementary Figure S5A and B). For Dol-16 the extrapolation to n=0 explains 40 ± 11 and 99 ± 2 % of the observed population for D-DX or D-DX/MVL, respectively. Interestingly, the effectiveness of incorporation of isoprene units derived from D-DX increases in the presence of MVL and this effect was not observed for Pren-11.

Upon supplementation with D-MVL (Supplementary Figure S5D) extrapolation to n=0 explains a very low fraction of the population of Dol-16 of the natural isotopic abundance containing only isoprene units originating from the MVA pathway. Besides the pool of natural isotopic abundance Dol-16 (n=0), an additional population of Dol-16 with a high number of deuteriated isoprene units (9-16) is observed; the dominant form of Dol-16 in this population is that built of 16 deuteriated isoprene units, i.e. derived solely from D-MVL. Upon co-supplementation with DX (the D-MVL/DX experiment) the pool of natural isotopic abundance and weakly deuteriated Dol-16 (n=1,2) increases while the population of Dol-16 molecules with high numbers of deuteriated isoprene units broadens substantially: under these conditions Dol-16 molecules with all possible numbers of deuteriated isoprene units are identifiable. Moreover, the population of fully deuteriated Dol-16, although still large, decreases upon D-MVL/DX labeling when compared to labeling with D-MVL alone. Thus the effectiveness of incorporation of isoprene units derived from each of the two deuteriated precursors, D-DX and D-MVL, increases in the presence of the natural isotopic abundance precursor of the other pathway (with the exception of the pool of Dol-16 with all 16 deuteriated isoprene units – it is decreased in the D-MVL/DX experiment compared to D-MVL alone).

Further, careful inspection of the data for D-MVL and D-MVL/DX labeling (Supplementary Figure S5D) shows that Dol-16 molecules containing 11 or 12 deuteriated isoprene units (indicating that 4-5 units must be of MEP origin) are overpopulated. Such an observation is in line with our previous data suggesting that the initial isoprene units of Dol molecules originate from the MEP pathway [5].

Summarizing, the model obtained here indicates the coexistence of two pools of Dol-16 in leaves: one natural isotopic abundance (n=0) and the other of mixed origin (n=16). While upon single-precursor feeding a high fraction of the natural isotopic abundance Dol-16 pool is derived from the non-deuteriated pathway (~60% of MVA-originating molecules upon D-DX labeling and ~90% of MEP-originating molecules upon D-MVL labeling), co-supplementation with the natural isotopic abundance precursor of the other pathway (in the D-DX/MVL and D-MVL/DX experiments) makes these separate pools of Dol-16 of the natural isotopic abundance undetectable, indicating that under these conditions the enzymatic pathway(s) capable of using both exo- and endogenous substrates predominates. It is worth noting that upon supplementation solely with exogenous D-MVL two separate pools of Dol-16 are observed, one derived only from exogenous D-MVL (n=16) and the other only from endogenous substrates (n=0) (Supplementary Figure S5D).

Dol-16 in the roots: D-DX labeling in the roots results in two populations of Dol-16 originating either mainly from endogenous substrates (n=0, 1, 2, …) or mainly from exogenous D-DX (n= …14, 15, 16) (Supplementary Figure S5E). These two populations are almost symmetrically distributed (n=0,1,2… vs. n=16,15,14…, Supplementary Figure S5E), clearly indicating that the pools of exogenous D-DX-derived and endogenous substrates are separated also in roots. In this case, however, precursors derived from exogenous D-DX (most probably IPP) can serve as the sole precursors for Dol synthesis. Still the existence of a minor pool of partially labeled

Dol-16 molecules shows that exogenous and endogenous substrates are to some extent simultaneously accessible to the Dol biosynthetic machinery. Co-supplementation with exogenous natural isotopic abundance MVL (D-DX/MVL) makes the distribution of highly deuteriated molecules much broader, while the distribution of weakly deuteriated molecules remains almost unaffected (Supplementary Figure S5E). This indicates that exogenous MVL and/or its metabolites (IPP?) are not accessible in the subcompartment(s) in which Dol synthesis from endogenous substrates takes place, while metabolites originating from both types of exogenous substrates (D-DX and natural isotopic abundance MVL) are being simultaneously used in another subcompartment. It is also worth noting that the pool of Dol-16 molecules containing 3-5 D-DX-derived isoprene units are overpopulated (n=3-5, Supplementary Figure S5E), and this effect is much stronger than that observed for Dol synthesis in leaves upon supplementation with exogenous D-MVL or D-MVL/DX (Supplementary Figure S5D).

Supplementation with exogenous D-MVL also results in two pools of different origin (Supplementary Figure S5F), however in this case the fraction of natural isotopic abundance Dol-16 (n=0) that is not synthesized *via* the MVA pathway is considerably lower (~10% of the pool of natural isotopic abundance Dol) than that derived from the MVA pathway upon D-DX feeding (~100%). In addition, the population of weakly enriched molecules is also very low clearly indicating that metabolites (e.g. IPP) derived from exogenous D-MVL, contrary to D-DX metabolites, do not penetrate to the subcompartment in which endogenous substrates are preferably used. Moreover, upon D-MVL labeling an increased population of Dol-16 molecules containing 10-13 isoprene units of MVL origin is observed (n=13-10; consequently, 3-6 isoprene units in their molecules must be of MEP origin) – this observation is consistent with the results of Dol-16 labeling with D-DX in the roots (n=3-5, Supplementary Figure S5E). Upon co-supplementation with exogenous natural isotopic abundance DX (the D-MVL/DX experiment) the molecules originating from this and other endogenous substrates (n=0) predominate while the pool of weakly-enriched Dol-16 (n=1-10) is hardly detectable and the pool originating solely from the MVA pathway (n=16) is almost absent (Supplementary Figure S5F). Interestingly, a slight increase in the population of Dol-16 molecules (n=13-15) is still observed suggesting the MEP-origin of 1-3 i.u. in their molecules.

Supplementary Figure S6. Competitive vs. single-precursor labeling of phytosterols in Arabidopsis leaves (left column) and roots (right column).

The deuteriation profile was calculated for campesterol (A), stigmasterol (B) and sitosterol (C) after feeding of plants with each of the deuteriated precursors alone (D-DX and D-MVL, lower section of each panel) or in the presence of the natural abundance precursor of the other pathway (D-MVL/DX and D-DX/MVL, upper section of each panel).

In each plot the deuteriation profiles calculated for the respective competitive and single labeling experiments are juxtaposed. Shown are representative results (n = 2).

The deuteriation levels calculated for each experiment are presented in Supplementary Table S2.

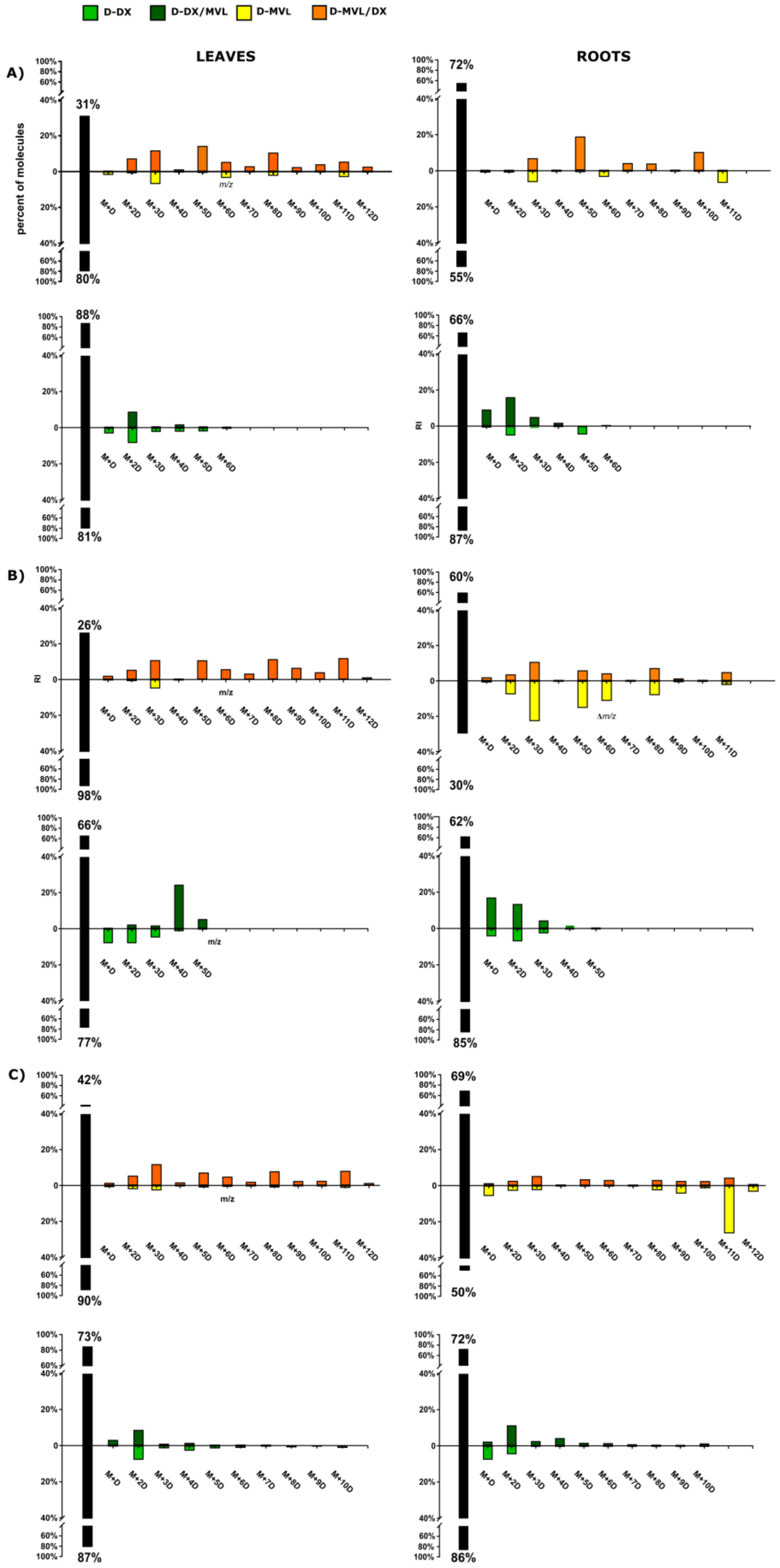
D-DX
D-DX/MVL
D-MVL
D-MVL/DX
A)
LEAVES
ROOTS
percent of molecules
RI
m/z
Δm/z
B)
C)
31%
80%
72%
55%
88%
81%
66%
87%
26%
98%
60%
30%
66%
77%
62%
85%
42%
90%
69%
50%
73%
87%
72%
86%

Supplementary Figure S7. Profiles of sterol precursors and phytosterols after feeding with D-DX, D-DX/MVL and D-MVL. Presented are GC/FID chromatograms of adequate lipid fractions isolated from the same amount of leaf (A) and root (B) tissue of labeled/control plants. For D-MVL labeling only leaf samples were available. CYCL: cycloartenol, 24-MCYCL: 24-methylene cycloartanol, CAMP-campesterol, STIGMA- stigmasterol, SITO- sitosterol.

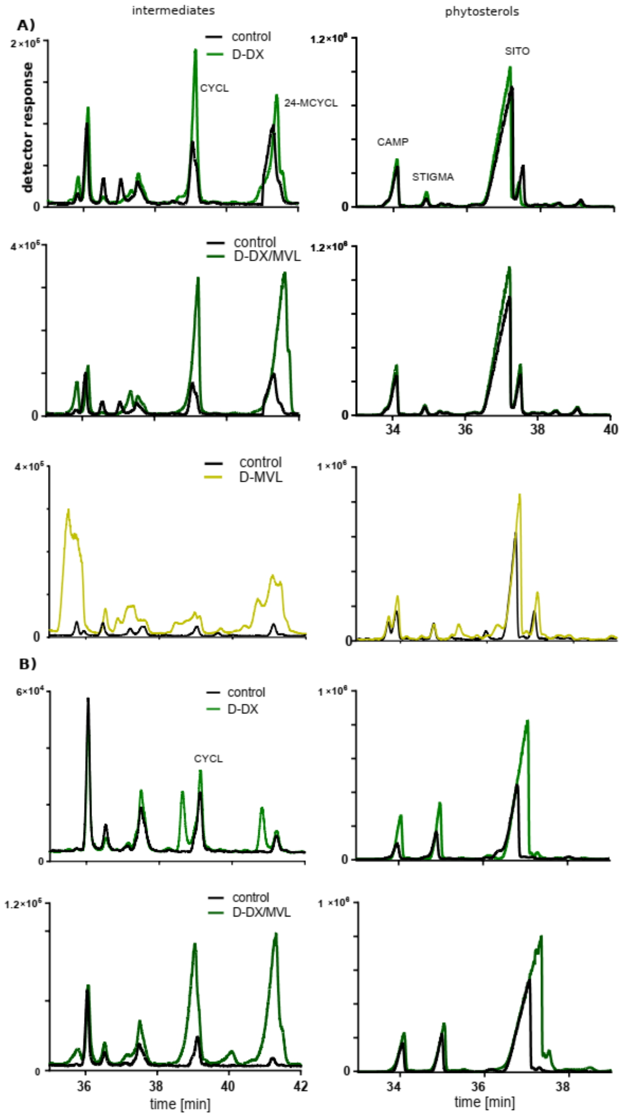

Commentary note to Supplementary Figure S7.

The profiles and accumulation levels of sterol precursors are clearly altered upon feeding with MVL (natural isotopic abundance or deuteriated). Lack of concomitant changes in the phytosterol profiles shows that rate-limiting enzymatic steps are present in the biosynthetic routes leading to plant sterols and that application of the exogenous precursor of the MVA pathway activates some feedback mechanism. Consequently, the deuteriation rates noted for phytosterols after D-MVL feeding might not accurately reflect the contribution of the MVA pathway since a fraction of the labeled precursor is most probably retained in the accumulated precursors.

Supplementary Figure S8. **LC/APCI-MS analysis of metabolically labeled lutein isolated from leaves of plants fed with various metabolic precursors.** Shown are full scale (left panels) and enlarged (right panels) fragments of mass spectra (note different scale of detector response). $[M+H-18]^+$ and $[M]^+$ ion species (*m/z* 551 and *m/z* 568) are marked by arrows. The M+3 signal is indicated in the spectra for the D-MVL and D-MVL/DX experiments.

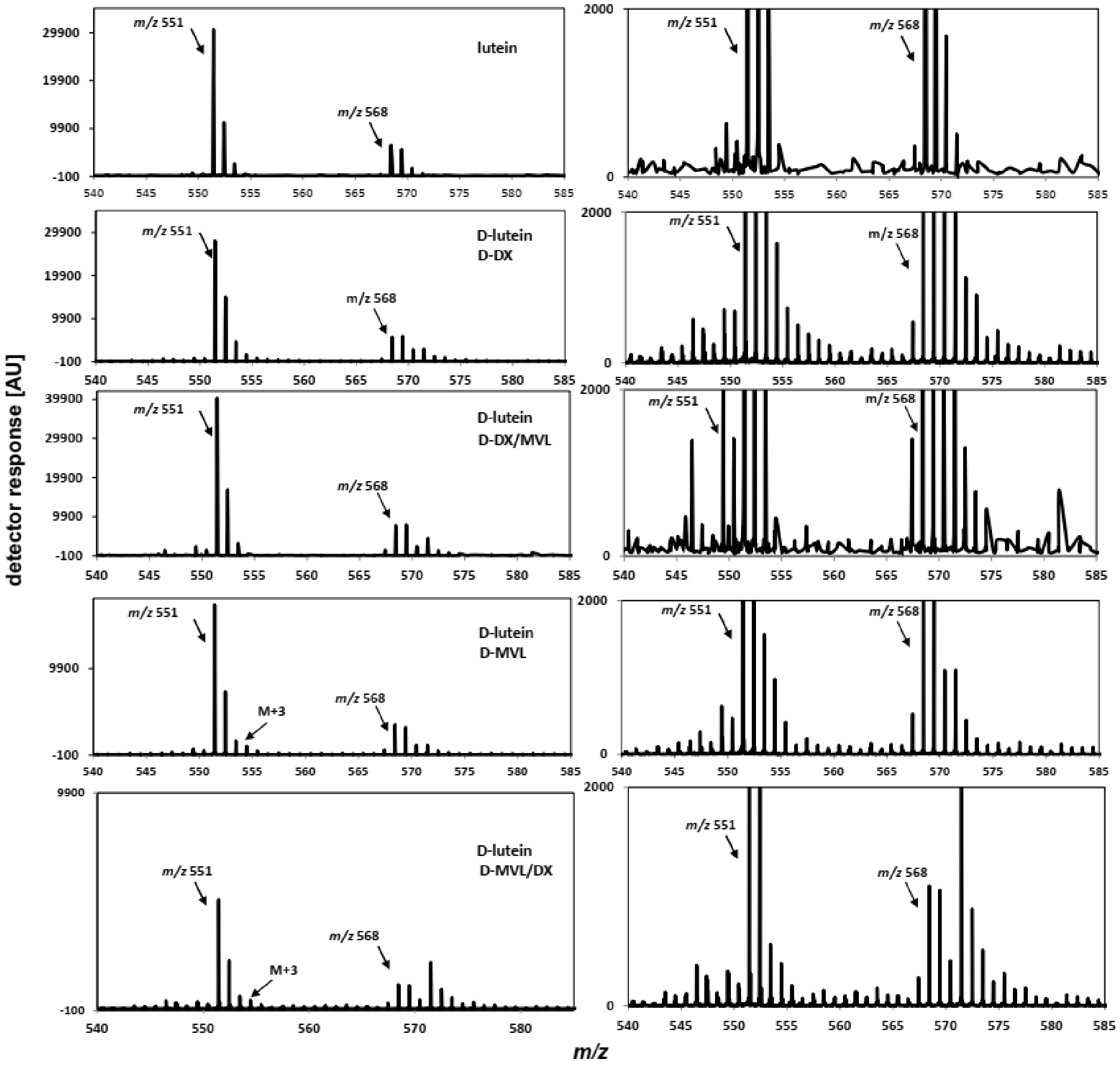

Commentary note to Supplementary Figure S8

The rate of metabolic turnover, a compound-specific feature, has to be taken into consideration when the results of long-term labeling are analyzed. A balance between intensive biosynthesis and degradation of carotenoids (which is in line with their photoprotective role) might be the reason for the low labeling efficiencies observed in this study compared to pulse-chase feeding of specific precursors in cotton seedlings (28). Indeed, $^{14}CO_2$-labeling revealed that carotenoids undergo continuous turnover even in mature Arabidopsis leaves [6]. The tight connection of plastidial pigment levels with photosynthesis and the strong dependence of their biosynthesis on assimilated CO_2 [7] might further contribute to their low deuteriation levels detected here.

Similarly, the low efficiency of polyprenol deuteriation upon feeding with D-DX might be affected by the rate of their turnover which has not been estimated for plants.

Supplementary Figure S9. **LC/APCI-MS analysis of metabolically labeled carotene isolated from leaves of plants fed with various metabolic precursors.** Shown are full scale (left panels) and enlarged (right panels) fragments of mass spectra (note different scale of detector response). $[M]^+$ and $[M+H]^+$ ion species (*m/z* 536 and *m/z* 537, respectively) are marked by arrows. An unidentified group of ion species abundant in the mass spectra of labeled carotene is also marked.

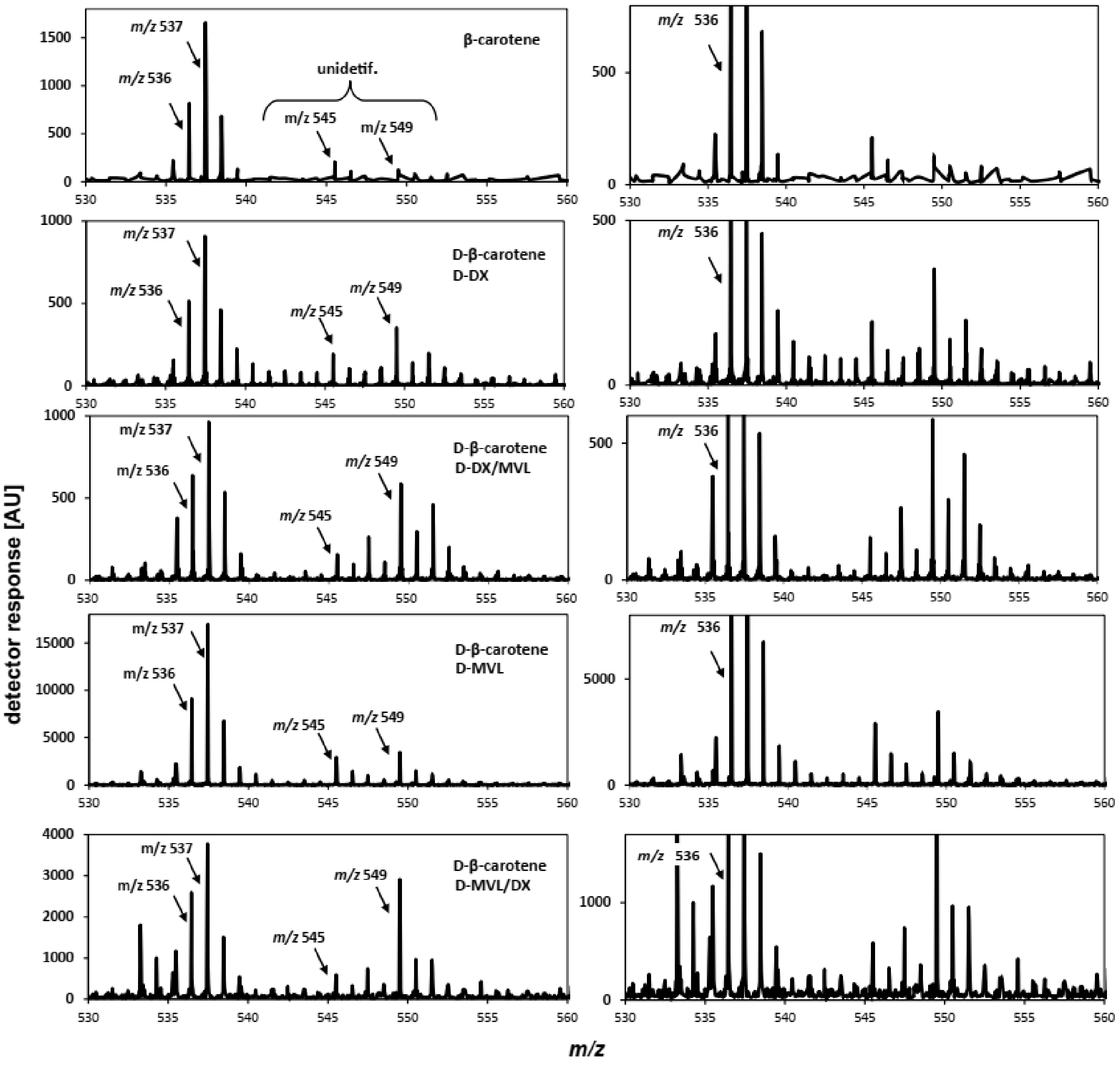

References

1 Popják, G. (1971) Specificity of enzymes of sterol biosynthesis. *Harvey Lect.* **65**, 127–156.

2 Nes, W. D., Campbell, B. C., Stafford, A. E., Haddon, W. F. and Benson, M. (1982) Metabolism of mevalonic acid to long chain fatty alcohols in an insect. *Biochem. Biophys. Res. Commun.* **108**, 1258–63.

3 Nes, W. D. and Bach, T. J. Evidence for a mevalonate shunt in a Tracheophyte. *Proc. R. Soc. London - Biol. Sci., Royal Society* **225**, 425–444.

4 Hemmerlin, A. (2013) Post-translational events and modifications regulating plant enzymes

involved in isoprenoid precursor biosynthesis. *Plant Sci.* **203**–**204**, 41–54.

5 Skorupinska-Tudek, K., Poznanski, J., Wojcik, J., Bienkowski, T., Szostkiewicz, I., Zelman-Femiak, M. et al. (2008) Contribution of the mevalonate and methylerythritol phosphate pathways to the biosynthesis of dolichols in plants. *J. Biol. Chem.* **283**, 21024–35.

6 Beisel, K. G., Jahnke, S., Hofmann, D., Köppchen, S., Schurr, U. and Matsubara, S. (2010) Continuous turnover of carotenes and chlorophyll a in mature leaves of Arabidopsis revealed by $^{14}CO_2$ pulse-chase labeling. *Plant Physiol.* **152**, 2188–99.

7 Wright, L. P., Rohwer, J. M., Ghirardo, A., Hammerbacher, A., Ortiz-Alcaide, M., Raguschke, B., et al. (2014) Deoxyxylulose 5-phosphate synthase controls flux through the methylerythritol 4-phosphate pathway in Arabidopsis. *Plant Physiol.* **165**, 1488–1504.
